# Combining HR-TEM and XPS to elucidate the core–shell structure of ultrabright CdSe/CdS semiconductor quantum dots

**DOI:** 10.1038/s41598-020-77530-z

**Published:** 2020-11-26

**Authors:** Florian Weigert, Anja Müller, Ines Häusler, Daniel Geißler, Dieter Skroblin, Michael Krumrey, Wolfgang Unger, Jörg Radnik, Ute Resch-Genger

**Affiliations:** 1 Division 1.2 Biophotonics, Federal Institute for Material Research and Testing (BAM), Richard-Willstätter-Str. 11, 12489 Berlin, Germany; 2 Division 6.1 Surface Analysis and Interfacial Chemistry, Federal Institute for Material Research and Testing (BAM), Unter den Eichen 44-46, 12203 Berlin, Germany; 3 Institut Für Optik Und Atomare Physik, Technische Universität Berlin, Straße des 17. Juni 135, 10623 Berlin, Germany; 4 Physikalisch-Technische Bundesanstalt (PTB), Abbestr. 2-12, 10587 Berlin, Germany

**Keywords:** Chemistry, Materials science

## Abstract

Controlling thickness and tightness of surface passivation shells is crucial for many applications of core–shell nanoparticles (NP). Usually, to determine shell thickness, core and core/shell particle are measured individually requiring the availability of both nanoobjects. This is often not fulfilled for functional nanomaterials such as many photoluminescent semiconductor quantum dots (QD) used for bioimaging, solid state lighting, and display technologies as the core does not show the application-relevant functionality like a high photoluminescence (PL) quantum yield, calling for a whole nanoobject approach. By combining high-resolution transmission electron microscopy (HR-TEM) and X-ray photoelectron spectroscopy (XPS), a novel whole nanoobject approach is developed representatively for an ultrabright oleic acid-stabilized, thick shell CdSe/CdS QD with a PL quantum yield close to unity. The size of this spectroscopically assessed QD, is in the range of the information depth of usual laboratory XPS. Information on particle size and monodispersity were validated with dynamic light scattering (DLS) and small angle X-ray scattering (SAXS) and compared to data derived from optical measurements. In addition to demonstrating the potential of this novel whole nanoobject approach for determining architectures of small nanoparticles, the presented results also highlight challenges faced by different sizing and structural analysis methods and method-inherent uncertainties.

## Introduction

**Figure 1:**
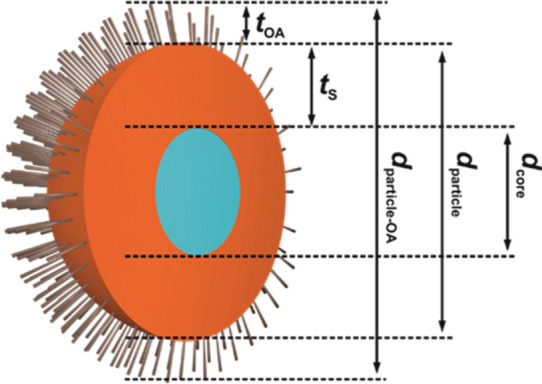
Model of the CdSe/CdS QD. *t*_OA_: thickness of the oleylamine ligand shell; *t*_S_ thickness of the CdS shell; *d*_core_: diameter of the CdSe core; *d*_particle_: diameter of the particle which is detectable with TEM and SAXS unequivocally; *d*_particle-OA_: diameter of the particle including the organic shell.

Surface chemistry is an increasingly relevant field in nanoparticle (NP)-related research and particularly relevant for many applications of nanomaterials^1,2^. Examples present nanometer-sized semiconductor quantum dots (QD) (see Fig. 1) with their size tunable optical properties and narrow emission bands with applications in display technology, solid state lighting, and solar energy conversion^3,4^. For optimum performance, i.e., a very high photoluminescence quantum yield (*Φ*_PL_) close to unity and an excellent photostability, the non-radiative recombination of charge carriers commonly associated with trap states must be suppressed^5–7^, typically with the aid of tight inorganic surface passivation shells of sufficient thickness^8–10^. This has meanwhile led to few reports of QD with *Φ*_PL_ close to unity for the best studied class of II/VI QD, either exploiting so-called “giant” CdSe/CdS core/shell quantum dots (g-QD)^11,12^, or a post-synthetic treatment with e.g. chloride ions^13^. These design concepts can also provide non- or barely blinking and very photostable QD, e.g. for bioimaging and single particle tracking studies^14,15^.

The crucial importance of passivation shell thickness control for QD with high *Φ*_PL_^15–17^ calls for reliable methods to assess particle architecture. Relevant features include size, shape, and size distribution of the core and core–shell nanostructure, as well as the thickness and chemical composition of the inorganic surface passivation shell(s)^18,19^. Also the chemical nature and number of stabilizing ligands per NP is relevant as they determine the colloidal stability, processability, e.g., the ease of incorporation into matrices like polymers for device fabrication, and (bio)functionalization^20,21^. Size and shape of core and core–shell nanostructures and insights into the particle architecture are commonly obtained with analytical techniques like (high resolution) transmission electron microscopy ((HR-)TEM), scattering techniques like small and wide angle X-ray scattering (SAXS and WAXS), and wavelength dispersive X-ray spectroscopy (WDX), as well as dynamic light scattering (DLS) and small-angle neutron scattering (SANS)^22^. Very small inorganic NP like QD with sizes below 10 nm are mainly analyzed with TEM, measuring dried samples on a substrate in vacuum^23^. Ideally, first the as-synthesized core is analyzed and then the whole core/shell nanostructure after deposition of the shell(s). The size difference between both nanoobjects is then taken as a measure for shell thickness. To obtain statistically significant results with this single particle technique, several hundred particles must be evaluated which is a general problem of TEM investigations^24,25^. Aside from relying on the availability of the core particles, core/shell intermixing or other unexpected deviations of the core/shell nanostructure like inhomogeneities of the shell cannot be detected.

Less frequently, X-ray diffraction (XRD) is used, also employing dried samples^23,26^. However, TEM and XRD provide only size information of the inorganic core, including the inorganic passivation shell(s), while size contributions from organic capping ligands containing light elements are missed. Alternatively, the size and size distribution of ensembles of dispersed NP can be obtained with scattering techniques like SAXS which evaluates the angular distribution of an X-ray beam scattered by nanoscale structures in the forward direction under small angles^27,28^ or by DLS^29^. These scattering techniques provide information on a large number of particles in a more application-relevant environment, offer better and easier accessible statistics compared to TEM, and can circumvent possible particle aggregation induced upon the drying of particle suspensions.

Principally, the simplest technique for determining the size of QD is the measurement of the size-dependent absorption spectra of QD dispersions. The spectral position of the first excitonic absorption peak is then correlated with QD size via so-called sizing curves that rely on size measurements of core-only nanostructures with TEM or XRD^23,30^. This approach is strictly valid solely for core-only systems, as the growth conditions of the shell can have a strong impact on the optical properties and the quantum confinement of the resulting core–shell nanostructures^31^.

An emerging method for analyzing the surface chemistry and the core/shell structure of NP presents X-ray photoelectron spectroscopy (XPS), especially for systems with low image contrast in TEM due to similar lattice constants of the core and the shell^32,33^. XPS measures the kinetic energy and number of the electrons that escape from the top of a sample in vacuum up to a penetration depth of 5–10 nm upon irradiation with an X-ray beam. This technique, which has been widely used for surface analysis of planar substrates and lately also microparticles, can provide information on the chemical composition and amount of chemical groups or certain chemical species at the particle surface^34^. Due to the sensitivity of XPS for lighter elements, not only inorganic surface coatings, passivation shells, and surface modifications by chemical processes like oxidation or etching can be detected^13,35,36^, but also organic coatings, ligands, and biomolecules, which are often not or only very hardly visible with other methods, e.g. TEM^37^. For small NP with sizes approaching the penetration depth of XPS, however, challenges such as surface curvature must be overcome for the reliable determination of the thickness of surface shells with the aid of models that consider particle size and surface curvature^25,38–40^.

In this study, we introduce a whole nanoobject approach for the analysis of the size and particle architecture of nanostructures, exemplarily for a thick shell CdSe/CdS QD with a *Φ*_PL_ close to unity and minimum blinking using (HR-)TEM, DLS, SAXS, and XPS. Special emphasis is dedicated to the determination of the core diameter and the shell thickness of this nanomaterial by combining sophisticated HR-TEM and XPS measurements and mathematical modeling of the data with the software SESSA (Simulation of Electron Spectra for Surface Analysis)^41^. The ultimate goal is to underline the potential of this method combination for the straightforward determination of core–shell structures, thereby circumventing individual measurements of core/shell NP and the initially prepared core often not available, particularly for commercialized core/shell nanostructures. In this context, we will also highlight challenges currently faced by the different sizing and structural analysis methods as well as uncertainties associated with the determination of the boundaries of the core/shell structure with HR-TEM and the effective attenuation length of the ejected electrons used for XPS.

## Experimental section

### Quantum dot synthesis

The synthesis of the thick shell CdSe/CdS QD stabilized with a mixture of oleic acid and oleylamine ligands was done following the method described by Chen *et al.* and Carbone *et al.* (plus an additional drying step)^14,42^ and published elsewhere^43^. The thick CdS shell was grown by slow continuous shell precursor infusion. In conjunction with the use of octanethiol, which has a relatively low reactivity, this yielded a slow and reproducible shell growth with good thickness control and monolayer precision. In Fig. 1 a schematic representation of the CdSe/CdS QD is shown.

### Absorption and emission spectroscopy

Absorption spectra of the oleylamine-stabilized CdSe/CdS QD dispersed in hexane were recorded on a calibrated Cary 5000 UV–Vis–NIR spectrometer (Varian, Agilent Technologies) with a spectral bandwidth and step size of 1 nm. The accuracy of the intensity and wavelength scale of this instrument is regularly controlled with certified absorption standards (Hellma GmbH). Steady state and time-resolved emission measurements were performed on a calibrated FLS920 fluorescence spectrometer (Edinburgh Instruments) equipped with a continuous xenon lamp (emission spectra) and a pulsed SC400-PP supercontinuum fiber laser (Fianium) with a pulse width of 0.1 ns using time-correlated single photon counting (TPSPC; fluorescence decay kinetics). So-called magic-angle conditions were applied (excitation and emission polarizers set to 0° and 54.7°, respectively) to render detected emission intensities independent of sample emission anisotropy^43^. The emission spectra were corrected for solvent emission (blank correction) and the wavelength dependence of the instrument’s spectral responsivity (spectral emission correction)^44^. The PL decay curves were fitted with a deconvolution fit using the software FAST (Edinburgh Instruments) using an instrument response function (IRF) determined with a Ludox silica particle dispersion as non-emissive scatterer. All optical measurements were done at room temperature (T = (22 ± 2) °C) in 1 cm quartz cells (Hellma).

### Absolute measurements of photoluminescence quantum yields

The *Φ*_PL_ values of the CdSe/CdS QD were determined absolutely with a calibrated integrating sphere setup Quantaurus-QY from Hamamatsu previously evaluated by us^45^ using long neck quartz cells from Hamamatsu. The absorbance at the excitation wavelength of 488 nm was kept below 0.1 to minimize inner filter effects and reabsorption which would lead to an underestimation of the Φ_PL_ values^44^.

### Single particle spectroscopy

For optical studies at the single particle level comparing the optical properties of our QD on a particle-to-particle level, a highly diluted hexane dispersion of the CdSe/CdS QD was spin-coated onto a glass cover slide. Single particle PL studies were performed with an inverted confocal laser scanning microscope, using a plan apochromatic oil immersion objective (100x, NA 1.4 by Olympus), mounted on a XYZ piezo, to scan the sample by moving the objective. For sample excitation, a 405 nm laser diode operated in the continuous or pulsed mode was employed. Two single photon sensitive avalanche photodiodes (SPAD by MPD), arranged in a Hanbury-Brown Twiss geometry, were used for the measurements of QD brightness, fluorescence intermittency via time traces (brightness over time), and fluorescence decay kinetics (data not shown). Single particle PL spectra were collected with an Ocean Optics Peltier-cooled CCD spectrometer (350–740 nm) coupled to the setup with an optical fiber. Discrimination of the excitation light and possible autofluorescence of the substrate was done with a dichroic mirror and long pass filters. The fluorescence intermittency of the single particles was obtained by recording the time traces at continuous excitation with the focus resting on the bright spots of the confocal image. The data presented were derived from measurements of more than 20 single QD.

### Transmission electron microscopy

Overview TEM images were obtained with a JEOL JEM-2100 (JEOL GmbH, Eching, Germany) operated at 200 kV. Image acquisition was done with a bottom-mounted 4 × 4 k CMOS camera (TemCam-F416, TVIPS, Gauting, Germany). Data analysis was performed with ImageJ and the particle size and size distribution were determined by averaging more than 2500 particles. The HR-TEM images were recorded with the FEI Titan 80–300 Berlin Holography Special TEM at 300 kV. This microscope is equipped with an image Cs-Corrector. To analyze the HR-TEM images with regard to the displacement field the ImageEval program^46^ was used. The analytical TEM images were acquired with a TEM/STEM JEOL JEM2200FS at 200 kV. The JEOL JEM2200FS is equipped with a LN2 free energy dispersive X-ray SD detector from Bruker for EDX measurements. A so-called beam shower was used whereby large areas of the grid were irradiated with a high dose electron beam for about 10 min. Thereby, amorphous carbon and other volatile impurities were removed from the peripheral area of the irradiated area where they formed a diffusion barrier. Additionally, a larger probe diameter of 1.5 nm was used to enhance the counting rates, which, however, slightly reduced the lateral resolution. The Bruker software Esprit was used for the evaluation and size analysis of the core/shell structure. The preparation of the TEM samples was done by placing one drop of the highly diluted QD dispersion on a carbon coated copper grid (200 mesh, Electron Microscopy Sciences, Hatfield, PA), which was then dried in air.

### Small-angle X-ray scattering

The scattering experiments were conducted at the four-crystal monochromator (FCM) beamline of PTB using the SAXS setup of the Helmholtz-Zentrum Berlin at the BESSY II synchrotron radiation facility^47^. At this beamline, any photon energy in the range from 1.75 keV up to 10 keV is available. To increase the contrast, SAXS measurements were performed at a photon energy of *E*_Ph_ = 3.42 keV close to the Cd L3 absorption edge. For the SAXS measurements, the QD were dried on a silicon nitride window with a thickness of only a few hundred nanometers. In addition, measurements were performed at *E*_Ph_ = 8 keV with a QD dispersion in hexane filled into a glass capillary. The scattered X-ray photons were collected on a vacuum-compatible PILATUS 1M hybrid-pixel detector (Dectris Ltd., Baden, Switzerland)^48^. The scattering intensity *I* is given as a function of the momentum transfer *q*(θ):

1 $$q\left(\uptheta \right)= \frac{4\uppi }{hc} {E}_{\mathrm{Ph}}\mathrm{sin}\theta$$

where *h* is the Planck constant, c the speed of light, *E*_Ph_ the energy of the photons and θ half of the scattering angle. For sufficiently monodisperse particle suspensions, the scattering curve *I(q)* shows pronounced oscillations, which depend on the particle diameter^49^. The scattering curves were fitted using the least-squares method as implemented in the program SASfit^50^ with a form factor for monodisperse solid spherical particles and a lognormal size distribution. The assumption of a spherical phase was supported by TEM studies. In addition, to account for particle aggregation, a structure factor for hard spheres in monodisperse approximation was used for the dried samples.

### Dynamic light scattering

The DLS experiments were carried out with a Zetasizer Nano instrument (Malvern Instruments, Worcestershire, UK) equipped with a 633 nm HeNe laser at a scattering angle of 173° (backscatter) using the “General purpose” analysis model and the default size analysis parameters. The QD dispersion was measured 12 times with ca. 15 runs per measurement and ca. 15 s per run (automatic default settings) in low volume quartz cuvettes. A refractive index of 2.29 for CdSe/CdS (calculated from the refractive indices of CdSe (2.59) and CdS (2.27) using the assumed volume fractions of core and shell) and an optical density of 0.1 were used as sample parameters for the conversion of the intensity-weighted to the number-weighted size distribution.

### X-ray photoelectron spectroscopy

A drop of a CdSe/ZnS QD dispersion in hexane was deposited onto a clean silicon wafer, previously treated with an UV Ozone Cleaner UVC-1014 (185 nm and 354 nm wavelength UV radiation source; manufactured by NanoBioAnalytics, Berlin, Germany) for 20 min, and dried in air. The sample was investigated with an AXIS Ultra DLD photoelectron spectrometer manufactured by Kratos Analytical (Manchester, UK) with monochromatic Al Kα radiation (*E*_Ph_ = 1486.6 eV) at a pressure of approximately 5 × 10^–9^ mbar. The electron emission angle was 0° and the source-to-analyzer angle was 60°. The binding energy scale of the instrument was calibrated following a Kratos Analytical procedure which uses ISO 15,472 binding energy data^51^. The XPS spectra were taken by setting the instrument to the hybrid lens mode and the slot mode providing approximately a 300 × 700 µm^2^ analysis area. Furthermore, the charge neutralizer was used. All spectra were recorded in the fixed analyzer transmission (FAT) mode. The intensities of the significant peaks were determined after subtraction of a Tougaard background using UNIFIT. For the simulation of the XPS spectra, the intensities of the peaks were corrected with the intensity-energy response function of the instrument.

### Simulation of electron spectra for surface analysis (SESSA)

The simulation of the XPS spectra was performed with SESSA v.2.0^41^. SESSA is a standard reference database distributed by the National Institute of Standards and Technology (NIST) that contains all data needed for quantitative simulations of XPS and Auger-electron spectra. Data retrieval is done with a powerful expert system that queries the databases and provides the data to the user or the simulation engine for arbitrarily shaped geometrical configurations. Individual photoelectron trajectories were simulated, and the resulting intensities were calculated from a statistically relevant large number of trajectories. The simulations were performed iteratively. The key quantities in this approach are the number of electrons arriving at the detector after a given number of collision number of inelastic collisions in the solid between the generation and the escape from the surface. The key parameter for the accuracy of the simulations is the effective attenuation length (EAL) which is determined with SESSA. The effective attenuation length corresponds to the inelastic mean free path, versus the average distance of an electron between successive inelastic collisions, corrected for the contribution of elastic scattering effects^52^. Inelastic mean free paths were obtained with the predictive TPP-2 M formula^53^ and elastic scattering cross sections were obtained from NIST Database SRD 64^54^. Depending on the physical model used for the calculations differences of up to 10% were observed^55,56^. The shell thicknesses were determined by finding the best match between the experimental and simulated data.

## Results and discussion

The main focus of this study was to quantitatively analyze the architecture of thick shell CdSe/CdS QD by combining HR-TEM and XPS in a whole nanoobject approach, that does not require measurements of the core NP, prior to shelling. For this purpose, we chose CdSe/CdS QD as a representative example as these II/VI QD are one of the best studied semiconductor nanocrystals relevant e.g. for solid state lightening and photovoltaics. Moreover, the fact that this core/shell nanomaterial contains solely Cd(II) ions as metal ions allows to reasonably estimate the thickness of the passivation shell from the preparation conditions and to minimize a possible influence of cation migration on the chemical composition of the shell. In the following, we present first the optical properties of these thick shell CdSe/CdS QD derived from ensemble and single particle measurements as a measure for particle quality. Subsequently, the determination of the particle size and morphology with DLS, SAXS, and TEM are shown. To elucidate the particle architecture and to quantitatively determine the core size and thickness of the passivation shell, we used two complementary methods, i.e., the combination of the single-particle microscopic method HR-TEM with the ensemble spectroscopic technique XPS. In the context of presenting our whole nanoobject approach, also the strengths and weaknesses of each method are discussed including a comparison of the uncertainties derived for all methods, thereby underlining the complementary insights gained from this method combination.

### Optical properties of the thick-shell CdSe/CdS QD

Prior to the structural analysis of the thick-shell CdSe/CdS QD their optical properties were assessed on the ensemble and single particle level to highlight their application potential. Moreover, the spectral position of the first excitonic absorption band, the spectral width or full width at half maximum (FWHM) of the first absorption maximum and the emission band, the *Φ*_PL_ and PL decay kinetics provide information on QD size, size distribution, and quality of the surface passivation shell. As shown in Figure S1 (left) in the Supporting Information (SI), the first excitonic absorption maximum and the emission maximum are located at 605 nm and 622 nm with FWHM of 15 nm (~ 51 meV) and 31 nm (~ 100 meV), respectively. These features closely match with the properties reported by Chen *et al.* for a thick-shell CdSe/CdS QD with a core diameter of about 4.2 nm, prepared with the same synthesis method as used for our sample, revealing a first excitonic absorption band at 607 nm, an emission maximum at 618 nm, and FWHM of the absorption and emission band of about 37 meV and 65 meV, respectively^11,14^. This suggests a similar core/shell size and similar structures of both QD and a slightly broader particle size distribution of our sample. The latter was also confirmed by single particle spectroscopic studies summarized in Figure S1 in the SI (dashed lines in the left panel) revealing variations in the spectral position of the emission maximum from 618 to 640 nm and a FWHM of the single particle emission band of ~ 12 nm (~ 36 meV). The very high *Φ*_PL_ of 95%, the quasi mono-exponential decay behavior (see SI, Figure S1, right), and the independence of Φ_PL_ on QD concentration derived from dilution studies covering 3 orders of magnitude (see SI, Figure S2) as well as blinking studies (see SI, Figures S3 and S4; QD fraction in the ON state of about 90%) underline the excellent surface passivation of our QD by the thick CdS shell. These findings point also to a rather homogeneous thickness of the surface passivation shell within the QD ensemble and to relatively small strain between the QD core and the shell.

As mentioned in the introduction, in principle, the size of QD can be derived from the spectral position of the first excitonic absorption maximum using empirical sizing curves determined for core-only QD^23^. However, the growth of a surface passivation shell can bathochromically shift the absorption maximum by several 10 nm depending on the reaction conditions used for the shelling step like temperature, reaction time, shell precursors etc^43,57^. These changes cannot be really considered by sizing curves and are difficult to predict by quantum mechanical modeling. Assuming that the CdS shell shifts the first excitonic absorption maximum to the red by about 20 nm (i.e., from hypothetical 585 nm for the core-only QD to 605 nm as found for the core/shell QD), the diameter of the CdSe core is estimated to 4.0 nm from these sizing curves. For the approach used for the shelling of the QD core, the thickness of the CdS passivation shell can also be estimated from the synthesis conditions such as the amount of the precursor and the growth time. As 11 monolayers of CdS were deposited onto the CdSe cores, each having a thickness of 0.337 nm^8^, the overall shell thickness is assumed to be approximately 3.7 nm, resulting in an overall QD diameter of about 11.4 nm.

### Size of the CdSe/CdS QD with scattering methods (SAXS and DLS)

**Figure 2:**
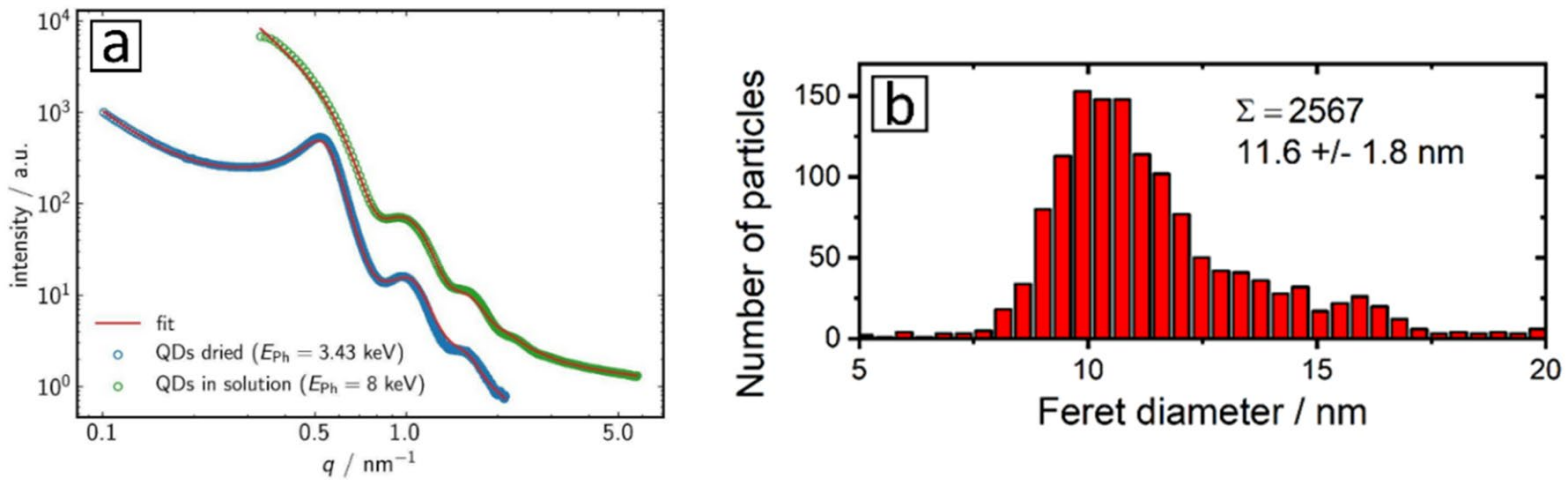
(**a**) SAXS scattering curves obtained for QD dried on a membrane (blue) and for QD dispersed in hexane (green) together with the fits of the curves. (**b**) QD size distribution from TEM. For the evaluation of the particle size and size distribution of the CdSe/CdS QD sample from the TEM images the software ImageJ was used.

Scattering methods like SAXS and DLS can provide fast information about the mean size of a large number of particles. Particularly the laboratory technique DLS is very frequently used for size measurements. In addition, the statistical basis of these methods is much better than that of TEM because a larger number of particles is assessed. The scattering pattern derived from SAXS measurements of our dispersed and dried QD show concentric rings with a diameter, that is directly related to the mean diameter of the particles *d*_particle_. The shape of the features (wiggles) in the SAXS curves provides a direct clue for the width of the particle size distribution. The pronounced oscillations in the scattering curves revealed in Fig. 2a underpin the narrow size distribution of the CdSe/CdS QD sample. SAXS measurements yielded a number weighted mean NP diameter of *d*_F_ = (10.9 ± 0.6) nm. The form factor-based diameters obtained for the dried and dispersed QD agree well within their stated standard uncertainties, which are dominated by the uncertainties of the data fitting procedure. Uncertainty contributions from the repeatability of the measurements and the input parameters (photon, energy, detector distance, detector pixel size etc.) are significantly lower. The distinct peak at *q* ≈ 0.5 nm^−1^ for the dried QD is ascribed to particle agglomeration, that is subsequently considered by overlapping the data with a structure factor. This leads however, to higher uncertainties. The NP diameter estimated from the hard sphere structure factor model (*d*_S_ ≈ 11.7 nm) is slightly higher than the form factor. This could be an indication for contributions from the organic ligand shell and for a particle shape which deviates slightly from a perfect sphere.

DLS measurements that provide the hydrodynamic diameter of the dispersed nanoobjects yielded a slightly larger particle diameter of about 14.8 nm with a standard deviation of 2.3 nm and a polydispersity index (PdI) of 0.28. The size distribution determined with DLS is shown in Figure S5 in the SI. Although DLS is one of the most frequently methods for particle size determination, it is often considered as being less precise because of the considerable impact of the measurement conditions and fitting parameters on the results. In addition, simple laboratory instruments can typically measure the intensity of the scattered light of the incident laser only at a single fixed angle, which is strictly suited only for the determination of the size of spherical objects. Moreover, in the case of strongly absorbing NP like QD, NP absorption of the laser light and NP PL can lead to a reduction of the data quality^29^. Also the conversion of the measured intensity-weighed size distribution into a number-weighed size-distribution, required to account for the more sensitive response of DLS to larger particles than to smaller ones^58^, can enhance the measurement uncertainty since it relies on several assumptions^59,60^.

### Size of the CdSe/CdS QD with TEM

TEM measurements, that present the most frequently used sizing method for small inorganic nanostructures, provide only information on the inorganic constituents, i.e., the core and the inorganic passivation shell *d*_particle_. The reliability of TEM size analysis depends largely on the parameters used for the TEM measurement and the image analysis (e.g., edge detection, aggregate and noise neglection, etc.). Moreover, a statistically meaningful number of particles, typically at least 500 particles, must be evaluated. For this study, we measured more than 2500 particles. The size histogram of all CdSe/CdS QD imaged are shown in Fig. 2b. These size measurements yielded a mean particle diameter of 11.6 nm with a standard deviation of 1.8 nm. This value agrees well with the diameter derived from SAXS measurements. It must be noted here that particle agglomeration during TEM sample preparation can lead to larger particle diameters, because not all single particles can be separated in the image. For further analysis with XPS shown in one of the following sections, we used the particle diameter obtained with TEM and SAXS. To account for eventual irregularities in particle shape, we exploit the Feret diameter, that equals the mean of the minimum and maximum distances between two parallel tangents to the contour of the particle (measurement by a slide gauge).

### Chemical analysis with STEM-EDX

A challenge of the TEM analysis of core/shell nanostructures can present the discrimination between the core and shell region, particularly for systems like our thick-shell CdSe/CdS QD, that consist of materials of similar crystal structures with only small differences in lattice constants. Therefore, different approaches were tested to locate the spatial position of the core within the core/shell nanostructure: Element mapping with Scanning TEM (STEM) and EDX as well as structural analysis with HR-TEM.

**Figure 3:**
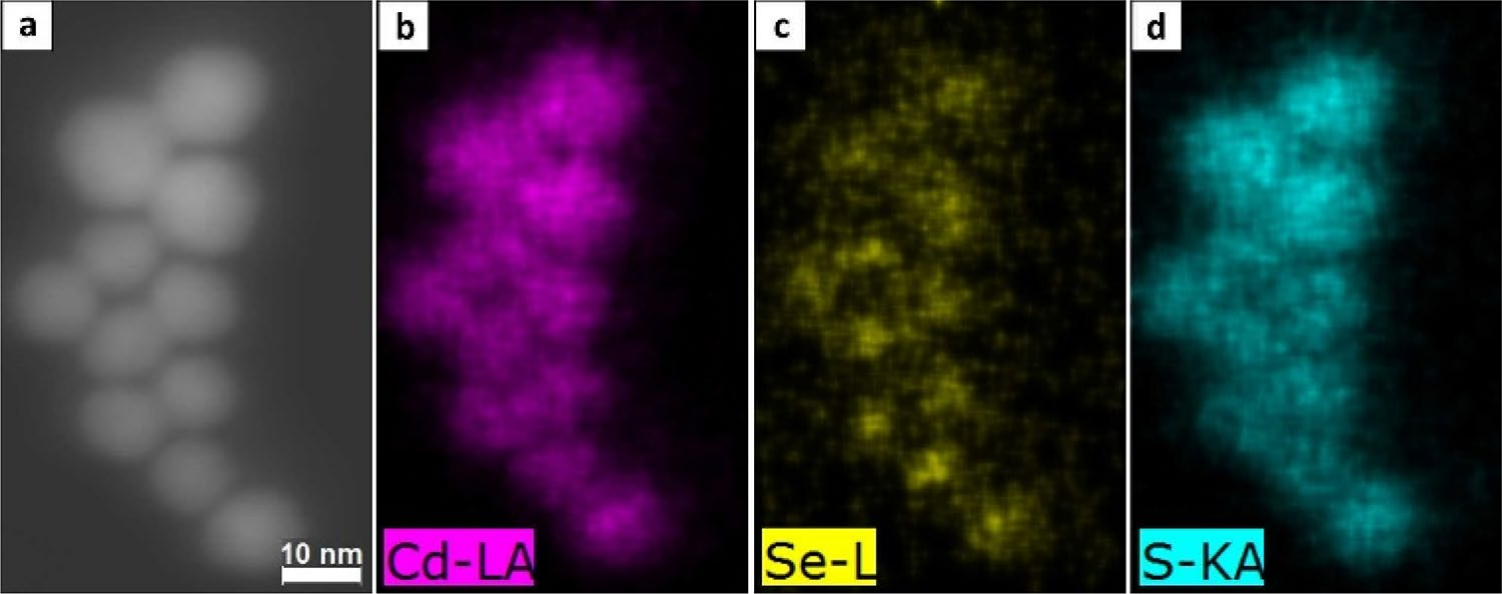
(**a**) STEM-EDX element mapping of the CdSe/CdS QD: HAADF image. (**b**) Cd-L α element map. (**c**) Se-L element map. (**d**) S-K α element map.

The most common method to determine the core position and shell thickness is element mapping. Challenges are the fast contamination with amorphous carbon and the small excitation volume which results in low counting rates of the EDX detector. To address the former, the experimental conditions were optimized as described before. Figure 3 shows a high-angle annular dark-field (HAADF) image with the spatially resolved EDX element distribution of cadmium (Cd), sulfur (S), and selenium (Se). The HAADF image (Fig. 3a) does not reveal a clear separation between core and shell. As expected, Cd is found in the entire particle (Fig. 3b), whereas Se is enriched in the core region (Fig. 3c). In contrast, S cannot be clearly located in the shell (Fig. 3d). This is attributed to the projection of the 3D particle shell onto the 2D TEM image. Although theoretically, a slight variation in S concentration can be expected between the edges and center of the CdSe/CdS QD. Here, the S containing surface passivation shell seems to be too thick in comparison with the core to observe such an effect. The EDX sum spectrum of the core regions (see SI, Figure S6) reveals a higher Se content than the sum spectra of the whole particle region (also shown in Figure S6) within the core/shell structure. Overall, we could verify a Se enrichment in the core with EDX, but a quantitative determination of the core and shell sizes was not possible with our instrumentation.

### Analysis of the core–shell structure with HR-TEM

**Figure 4:**
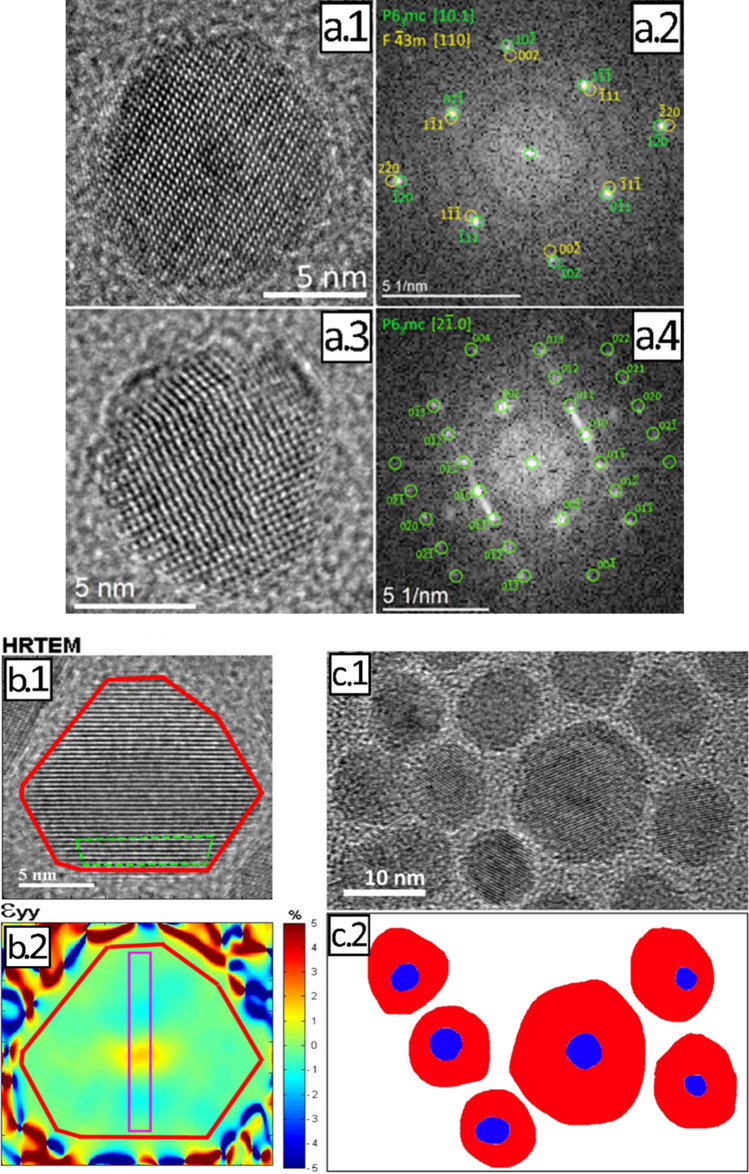
Results of the analysis of the core–shell structure with HR-TEM. (**a**) Determination of the crystal structure based on the analysis of the HR-TEM images of individual CdSe/CdS particles (shown in panels (**a.1**) and (**a.3**)) and the resulting Fourier transformations (FTs), shown in panels (**a.2**) and (**a.4**), respectively, superimposed with the corresponding simulated diffraction pattern. The software jems^62^ was used for the simulation of the diffraction patterns. (**b**) Geometric phase analysis (GPA) of a HR-TEM net plane image (**b.1**), where the analyzed area is marked in red and the reference region is marked in green; the results of the GPA (**b.2**) reveal a maximum misfit of 2.7% in the core region. (**c**) HR-TEM procedure used for determining the overall particle size, core size, and shell thickness: Defocused HR-TEM image revealing the core within the core/shell nanoparticle structures (**c.1**); Marked core areas (blue) and marked shell areas (red) used for data evaluation with the Bruker software (**c.2**).

Because it was not possible to obtain the desired information with element mapping, we assessed structural differences between core and shell by HR-TEM. Both, the CdSe core and the CdS shell can either occur in the cubic zinc blende or the hexagonal wurtzite structure^61^. To clarify the crystal phases of core and shell, the HR-TEM images of the CdSe/CdS QD and their corresponding Fourier transformations (FT) were evaluated and compared with the simulated diffraction images of the hexagonal wurtzite (space group: P63mc) and the cubic zinc blende phase structures (space group: F-43 m) as shown in Fig. 4a.1–4. The best agreement was found for a hexagonal structure of both CdSe and CdS. The similar crystal structures together with the small difference in lattice constants of CdSe and CdS explain why the differentiation between core and shell structure is challenging.

A usual approach to distinguish between core and shell is to investigate, whether a displacement of the atoms in the shell or at the core–shell interface from the ideal position can be found, that originates from strain caused by epitaxial growth. Therefore, we determined the position of the local maxima of the HR-TEM interferences (atomic column) relative to the respective reference lattice (see SI, Figure S7). As a reference, the maxima of the inner core region (core area marked in green in Figure S7, left) was used. The results allow, however, no clear distinction between the core and shell. The observed larger displacement at the edges results from the change in thickness of the illuminated regions of the QD particle.

Another approach to detect strain and deformation in HR-TEM images is geometric phase analysis (GPA). Thereby, variations in the periodicities of the HR-TEM contrast in different regions of the nanoobject are analyzed. A maximum misfit of 2.7% was detected between the whole particle and a reference region (Fig. 4b and Figure S8 in the SI). The results were very similar, independent of the chosen reference region, i.e., the shell or the core. This value is significantly lower than the result of 4.2% reported for CdSe/CdS nanorods^42^. These differences are ascribed to the different shape of both systems.

Overall, a high structural coherence between core and shell was found. This is beneficial for QD stability and a high Φ_PL_ but renders the analysis of the structural parameters challenging. The best results for distinguishing between the core and shell region were obtained with slightly defocused high-resolution TEM images (see Fig. 4c.1), exploiting Fresnel diffraction^63,64^. Thereby, a better phase contrast was detected than in the exact focus position. With this approach, it was possible to evaluate the aspect ratio of 245 particles, that provides the shape, average particle size, and (x, y)-positions of the center of gravity. For this purpose, the core and shell regions were marked (see Fig. 4c.2). Only particles were used which allowed for a clear differentiation between the core and shell region. Critical for this procedure was the position of the particle slightly out of focus.

**Figure 5:**
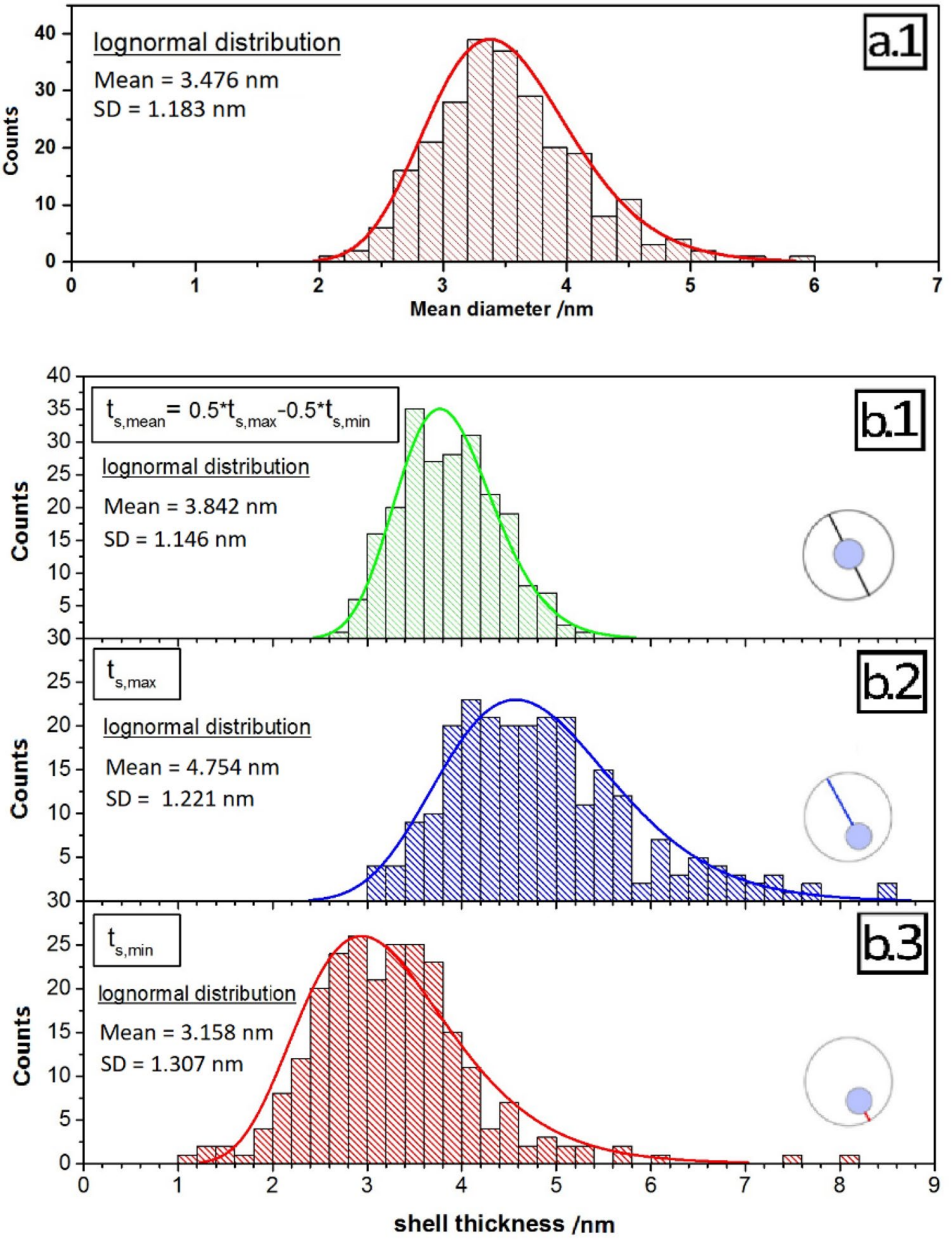
(**a.1**) Size distribution of the cores as derived from HR-TEM. (**b**) Statistics of the shell thickness of the CdSe/CdS QD: average shell thickness *t*_s,mean_ (**b.1**), maximum shell thickness *t*_s,max_ (**b.2**) and minimum shell thickness *t*_s,min_ (**b.3**).

The resulting aspect ratio of about 1.2 (see SI, Figure S9a) suggests slightly elongated cores. The lognormal size distribution of the cores has a maximum of 3.5 nm with a standard deviation (SD) of 1.2 nm as displayed in Fig. 5a. The aspect ratio of the complete core/shell particles, which have a mean diameter of 11.5 nm with a standard deviation of 1.2 nm (Figure S9c), amounted to about 1.1 (Figure S9b). This agrees very well with the SAXS results and the TEM studies of the particle size distribution. As follows from the comparison of the aspect ratios of the QD cores (Figure S9a) and the core/shell particles (Figure S9b) the growth of the thick CdS shell makes the particles slightly more spherical, which supports a uniform shell thickness.

Finally, the maximum and minimum shell thickness were determined from normal distributions (see SI, TEM section and Figure S10), and histograms shown in Fig. 5b.2,b.3. The difference between these shell thicknesses was determined to 1.6 nm. With an average size of the core/shell QD of 11.5 nm, this confirms the central position of the cores within the particles and a uniform shell growth. The derived histogram of the mean shell thickness (see Fig. 5b.1) shows two local maxima. The main maximum at 3.8 nm ± 1.1 nm reflects the mean shell thickness. The second maximum of 5.5 nm is ascribed to a very small contribution from larger NP. In summary, our detailed HR-TEM analysis reveals nearly spherical CdSe/CdS QD with a diameter of 11.5 nm, a centered core of 3.5 nm and a homogeneous shell with a thickness of 3.8 nm. The uncertainties amount to 1.2 nm for the core diameter and 1.1 nm for the shell thickness, respectively.

In the next section these results are compared with sizes obtained from XPS measurements. Such an approach of a mutual validation of quantitative results with two different complementary techniques allows to verify the obtained results. As with XPS no information about the size of the whole nanoparticle is accessible, in contrast to HR-TEM, we used the previously derived results of SAXS measurements of *d*_F_ = (10.9 ± 0.6) nm that agree well with the TEM data.

### Analysis of the CdSe/CdS QD with XPS

In the last years, an increasing number of examples for the potential of XPS to determine the elemental distribution in small inorganic NP and the chemical surface modification of semiconductor and lanthanide-based upconversion nanocrystals by light-induced changes, ligand exchange or the presence of certain species like chloride ions has been reported^13,30^. The characterization of the shell structure and thickness of core-(multi)shell QD by XPS is, however, challenging and requires sophisticated methods of data simulation. One approach to obtain NP size parameters presents the XPS tool SESSA^41^. Indispensable for this approach are information about the particle size and shape, which can be derived with different structure-analytical methods as described in the previous sections. Ideally suited for such studies are NP with a size in the range of the XPS information depth. In this case, all NP regions can be assessed and, simultaneously, the signals from particles below the upper NP layer are minimum. Therefore, a validation of the results obtained by XPS with the complementary TEM data is possible, even for such a rather sophisticated nanostructure with a low image contrast between core and shell in the TEM images as the CdSe/CdS QD.

**Figure 6:**
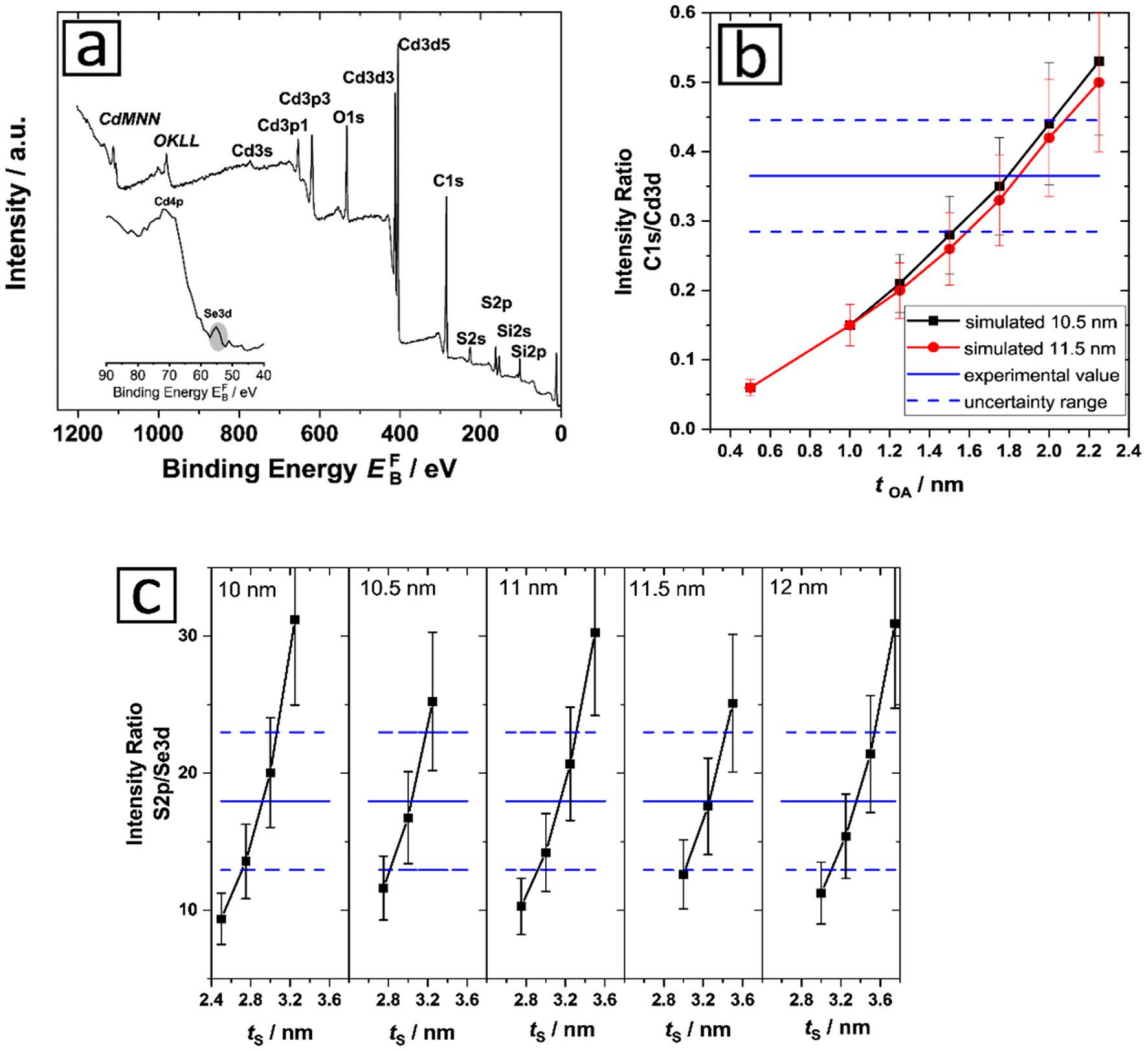
Results of the analysis of the CdSe/CdS core/shell QD with XPS. (**a**) Survey spectrum of the QD obtained with XPS. The inset shows the region between 90 and 40 eV. (**b**) Simulated ratio of the intensities of C 1 s and Cd 3d peaks for different thicknesses of the ligand shell *t*_OA_. According to the TEM and SAXS results particle diameters *d*_particle_ of 10.5 nm and 11.5 nm were assumed. The experimental value obtained from the XPS survey spectrum is marked with a blue line with an uncertainty range (confidence value of 95%) marked with the upper and lower blue line. (**c**) Simulated intensity ratios S 2p/Se 3d for different particle diameters between 10–12 nm as indicated by different thicknesses of the CdS shell. The experimental intensity ratio obtained from the XPS spectrum is given with lines revealing the respective uncertainty range (confidence level of 95%).

The XPS survey spectrum of the CdSe/CdS QD in Fig. 6a reveals peaks originating from the elements Cd, Se, and S constituting the QD as well as additional peaks, that point to the presence of Si, O, and C. Also, high-resolution spectra of all elements were recorded (see SI, Figure S11). Si and O are attributed to the Si wafer on which the QD were deposited. C is ascribed to the surface ligand oleylamine. The contribution of the Si wafer to the C signal is negligible (see SI, Figure S12). The ligand contribution is subsequently considered only for the simulation of the XPS results. Nitrogen as a further ligand component could not be detected due to the overlap of its signal with the dominating Cd 3d 5/2 peak. A significant influence of N1s on the Cd 3d5/2 peak can be excluded, because the ratio of the Cd 3d 5/2:3d 3/2 peaks is around 1.3, which is slightly lower than the ratio of 1.5 reported for metallic Cd^65^. This conforms with the low amount of N in the ligand and, herewith, in the whole investigated system.

The ratios of the signal intensities C 1 s/Cd 3d and S 2p/Se 3d (see Table S1) were determined to 0.37 and 17.96 with a maximum uncertainty (confidence interval of 95%) of about 10% after correction of the measured data with the spectrometer-dependent intensity-energy response function. For Cd 3d, the sum of both peaks, 3d 5/2 and 3d 3/2 were used. The intensity-energy response function was earlier obtained with a procedure previously described by Seah *et al.*^66^ and Hesse *et al.*^67^ This intensity-energy response function presents the main contribution to the uncertainty of the experimental intensity ratios. Other factors like the determination of the peak area or the repeatability were found to be less prominent.

For the subsequent analysis of the data derived from the XPS measurements of the CdSe/CdS NP, we assumed a layered sphere morphology of our QD with a CdSe core, a CdS surface passivation shell, and an outer oleylamine (C_18_H_35_NH_2_) ligand shell. The input parameter are shown in the SI in Table S2. A possible intermixing of the different layers was not considered although core–shell intermixing cannot be excluded^14,68^. This assumption is reasonable within the context of the uncertainty of the XPS measurements, which is in the order of ± 0.7 nm (equaling ± 2 ML CdS) as shown in the following section, and other publications on related CdSe/CdS QD, which refer to a thickness of a mixing layer of one or two ML^68,69^. For the modeling of the data, a single-sphere approximation was used which is valid for powders and random NP aggregates^70^. The assumption of spherical particles was justified by HR-TEM. The uncertainty of these results can be affected by factors like an inhomogeneous shell thickness and uncertainties in the effective attenuation length of the photoelectrons. For example, an inhomogeneous shell with a core displaced from the particle center can lead to an underestimation of the shell thickness^71^. For our core/shell NP, this was excluded by a detailed statistical analysis of the HR-TEM images. Therefore, the main uncertainty contribution arises from the uncertainty of the effective attenuation length of the photoelectrons; in a recent study combining X-ray reflection and XPS, an uncertainty of about 17% was determined for the effective attenuation length of Si 2p and Hf 4f electrons^67^. We estimated similar values for the photoelectrons considered in this study.

The analysis of the organic ligand shell of NP is challenging as it consists mainly of light elements like carbon and hydrogen which cannot be detected with TEM. In a very simplified model it is assumed, that the distance between densely packed adjacent NP equals twice the size of the ligand shell. The preparation of the TEM samples is very crucial for this method. It must be ensured that the sample displaced on the grid contains only a homogeneous layer of closely packed NP. Furthermore, to measure a large number of particles as required for sufficient statistics is a general challenge of TEM. Moreover, in this simple model only geometric factors are considered and other interactions like electrostatic ones or hydrogen bonding are neglected. In contrast, XPS enables to directly assess the organic ligands by measuring the C 1 s peaks and other components of the organic ligand shell. In accordance with this aim, the C 1 s/Cd 3d ratios were simulated for a QD with a diameter *d*_particle_ of 10.5 nm and 11.5 nm as derived from TEM and SAXS measurements. C and Cd were used for this simulation because the main component of the organic ligand is C (except for H which is not detectable with XPS) and the main component of the QD is Cd which is present in the shell and the core. In this approach the amount of adventitious carbon must be considered which is rather challenging. Based on our experiences, the amount of this carbon contamination was estimated to 10%. This number was then included in the measurement uncertainty as shown in Fig. 6b. The C contamination on the Si wafer was below 2 at%, and thus, can be neglected, as confirmed by measurements of a clean wafer (see SI, Figure S12). Comparing the value obtained experimentally for the C1s/Cd3d intensity ratio of 0.37 (Fig. 6b, blue line) with the simulated intensity ratios leads to a thickness of the organic ligand shell between 1.5 nm and 2.2 nm. Surprisingly, changes in the size of the QD between 10.5 nm and 11.5 nm influences the results only negligibly. This result agrees well with the rough estimation of the thickness of the organic ligand shell of about 2 nm, assuming a complete surface coverage by a ligand monolayer in a stretched conformation^72,73^. Additionally, the difference in particle size obtained with TEM and/or SAXS (except for the ligand shell) and the particle size determined with DLS (including the ligand shell) can provide a measure for the size of the ligand shell itself. This yields a shell thickness of 1.6 nm. For all these assumptions, the high flexibility of the ligand shell and the strong influence of the preparation method must be considered. The similarity of the results obtained with these simulations are notable although the XPS simulation was performed for the results obtained with dried QD samples and the DLS measurements with dispersed NP. This accounts for the resulting relatively high uncertainty.

With the information on the lower and upper NP size derived from the TEM and SAXS measurements it was possible to simulate the core–shell structure of our CdSe/CdS. As a surface analytical method XPS is more sensitive to the coating of nanoparticles, but with an overall information depth of XPS of about 10 nm it is possible to measure the whole NP. In Fig. 6c the results are shown that were obtained for a particle size varied between 10 and 12 nm. The experimental value derived for the S 2p/Se 3d peak ratio was determined to 18.0 from the respective XPS spectrum (see Fig. 6c, blue line). This value was compared with the simulated results derived for different particle sizes and shell thicknesses. For 10 nm- and 12 nm-sized particles, the simulated results yield shell thicknesses between 2.7 nm and 3.1 nm as well as 3.1 nm and 3.5 nm (including the corresponding measurement uncertainty), respectively. For the CdSe core, diameters between 4 nm and 5.8 nm were found. The accuracy and reliability of the determination of the shell thickness and the core size depends clearly on particle size, i.e., the size of the whole nanoobject used. With the knowledge of the exact particle size the core–shell structure of small NP with sizes of about 10 nm can be characterized with XPS with a high precision and relatively little experimental effort. The thickness of the carbon shell ascribed to the organic surface ligands influences the determination of the core and shell size only slightly (see SI, Table S3).

**Figure 7:**
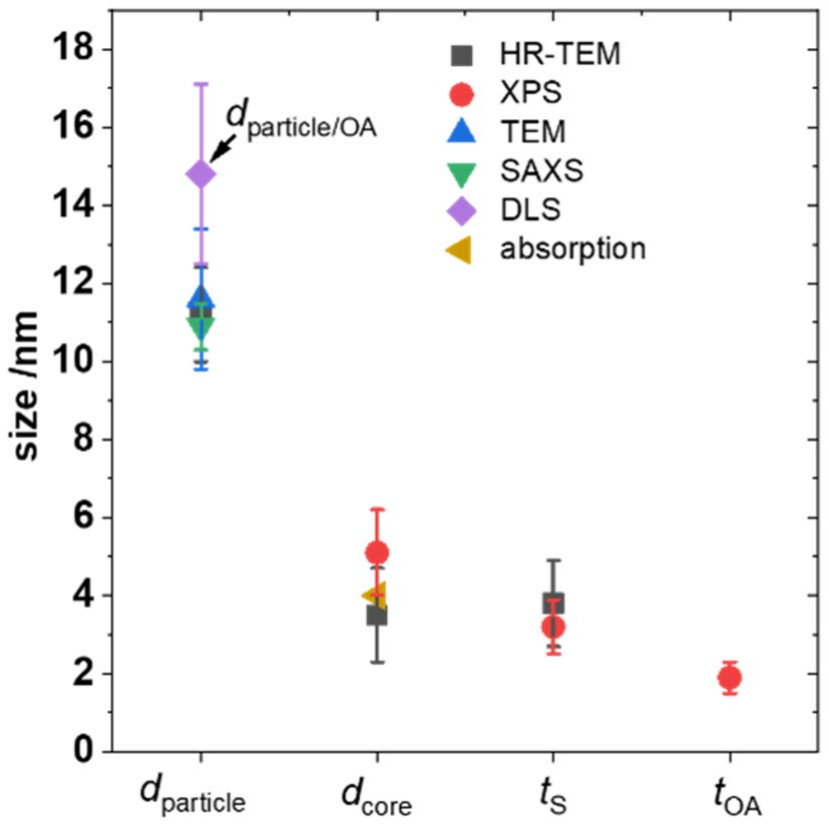
Summary of the different size results with uncertainties for the diameter of the particle including the inorganic shell (*d*_particle_) which is detectable with TEM, HR-TEM and SAXS unequivocally. DLS additionally considers the oleylamine ligand shell and herewith, the resulting particle diameter equals *d*_particle-OA_. The diameter of the CdSe core (*d*_core_) and the thickness of the CdS shell (*t*_S_) are both assessible with XPS and HR-TEM while the thickness of the oleylamine ligand shell (*t*_OA_) can be obtained only with XPS. The diameter of the core was estimated from the spectral position of the first excitonic absorption maximum which was taken from the absorption spectrum of oleylamine-stabilized CdSe/CdS QD shown in the SI in Figure S1.

A still open question in this study remains a possible intermixing between core and shell (see SI, Figure S13) as discussed for many core/shell nanostructures^74–76^. Due to the structural coherence between CdSe and CdS, a sharp boundary between core and shell was not observed with HR-TEM; and the lateral resolution of elemental mapping is not sufficient to provide this information. Among other factors, the probability of such effects depends on the synthetic method used for particle preparation. For example, nanomaterials synthesized at very high temperatures can be prone to cation migration^14,68,76^. Generally, inhomogeneities of the shell like core/shell intermixing is expected to lead to a smaller thickness due to the exponential character of the attenuation of the electrons. Comparing the HR-TEM with the XPS results reveals a slightly thinner shell for the XPS than the HR-TEM measurements, which could be a hint for core/shell intermixing. It must be, however, noted, that the observed deviation between both methods is within the uncertainty range of the two methods (see Fig. 7). A suitable method to clarify this question of core–shell intermixing present most likely XPS measurements with different excitation energies which were beyond the scope of the presented study.

## Conclusion and outlook

In summary, we demonstrated the potential of a combination of HR-TEM and XPS for the reliable determination of the core–shell structure of sophisticated nanoparticles (NP) like semiconductor quantum dots (QD) with sizes in the range of the information depth of XPS in a whole nanoobject approach. With this approach it is possible to obtain quantitative data on the particle architecture of such core/shell nanostructures without the need to measure the particles at different stages of their preparation, i.e., core formation and shelling with a surface protecting inorganic shell. Reliable particles sizes were obtained with sizing techniques like TEM, SAXS, and DLS, as well as from the position of the first excitonic maximum in the QD absorption spectrum, despite the discussed assumptions underlying the latter approach for core/shell QD. It must be noted that only the use of complementary techniques as presented here allow the mutual evaluation of each method.

The good match between the data obtained with these different methods is summarized in Fig. 7. The uncertainties reflect the method-inherent challenges associated with the determination of the boundaries of the core/shell structure with HR-TEM and the intensity-energy response function and the effective attenuation length of the ejected electrons for XPS. This also underlines the large potential of method combinations together with a mutual evaluation for a deeper understanding of the structural features of core/shell nanoobjects. Furthermore, inhomogeneities of the shell can be detected by combining X-ray photoelectron spectroscopy and electron microscopy which was recently shown for polymer particles^71^. In the future, we will expand this whole nanoobject approach to other application-relevant NP like lanthanide-doped upconversion nanocrystals of different size with different surface coatings.

## Supplementary information

Supplementary Information.
