# Supplementary material for: Combining HR-TEM and XPS to elucidate the core–shell structure of ultrabright CdSe/CdS semiconductor quantum dots
Source: Sci Rep. 2020 Nov 26;10:20712. doi: 10.1038/s41598-020-77530-z (PMC7692488; doi:10.1038/s41598-020-77530-z)
Supplement: Supplementary file 1 — Supplementary Information. [file 41598_2020_77530_MOESM1_ESM.docx]

**Supporting Information**

Combining HR-TEM and XPS to Elucidate the Core-Shell Structure of ultrabright CdSe/CdS Semiconductor Quantum Dots

Florian Weigert,^a^ Anja Müller,^b^ Ines Häusler,^c^ Daniel Geißler,^a^ Dieter Skroblin,^d^ Michael Krumrey,^d^ Wolfgang Unger,^b^ Jörg Radnik*^b^ and Ute Resch-Genger*^a^

1. Federal Institute for Material Research and Testing (BAM), Division 1.2 Biophotonics, Richard-Willstätter-Str. 11, 12489 Berlin, Germany. E-mail: ute.resch@bam.de
2. Federal Institute for Material Research and Testing (BAM), Division 6.1 Surface Analysis and Interfacial Chemistry, Unter den Eichen 44-46, 12203 Berlin, Germany. E-mail: [joerg.radnik@bam.de](mailto:joerg.radnik@bam.de).
3. Technische Universität Berlin, Institut für Optik und Atomare Physik, Straße des 17. Juni 135, 10623 Berlin, Germany
4. Physikalisch-Technische Bundesanstalt (PTB), Abbestr. 2-12, 10587 Berlin, Germany.

**Content:**

Figure S1. Emission spectra of a dilution series of the QD sample for stability testing

Figure S2. Confocal image of single OLA-stabilized CdSe/CdS QDs

Figure S3. Size histogram from DLS

Figure S4. Chemical analysis of the CdSe/CdS QDs with STEM-EDX

Figure S5. Determination of the coherence and tension of the CdSe/CdS nanoparticles

Figure S6. Core-shell structure

Figure S7. Determining the shell thickness

Figure S8. High-resolved photoelectron spectra

Figure S9: Survey spectrum with quantification of the pure UV/ozone cleaned Si wafer

Figure S10: Intermixing

Table S1. Corrected peak areas of the nanoparticles obtained with XPS

Table S2. Input Parameters for SESSA

Table S3. Influence of different ligand shell thicknesses d_OA_ on the S2p/Se 3d ratio

Table S4: Influence of different kind of ligand on the S2p/Se 3d ratio

**Figure S1.** *Left:* Emission spectra of a dilution series of the QDs after excitation at 405 nm. The integrated emission intensities (integration range 620-640 nm) displayed in the inset show a linear relationship with the QD concentration, demonstrating the independence of the PL QY on QD concentration in a concentration range covering three orders of magnitude. *Right:* The intensity-normalized emission spectra of the QD dilution series show no spectral shifts, underline the excellent surface passivation of the QD by the thick CdS shell.


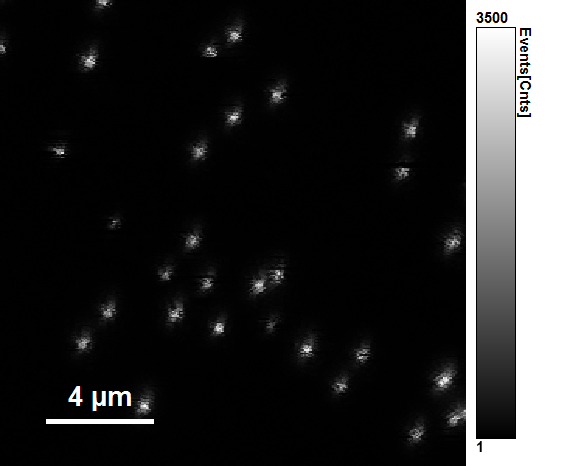


**Figure S2.** Confocal image of single OLA-stabilized CdSe/CdS QDs spincoated on a coverslip and measured with a scan range of (17 x 17) µm (at 300 pixels per line and 5 ms integration time per pixel) under continuous laser excitation at 405 nm (3.06 eV).


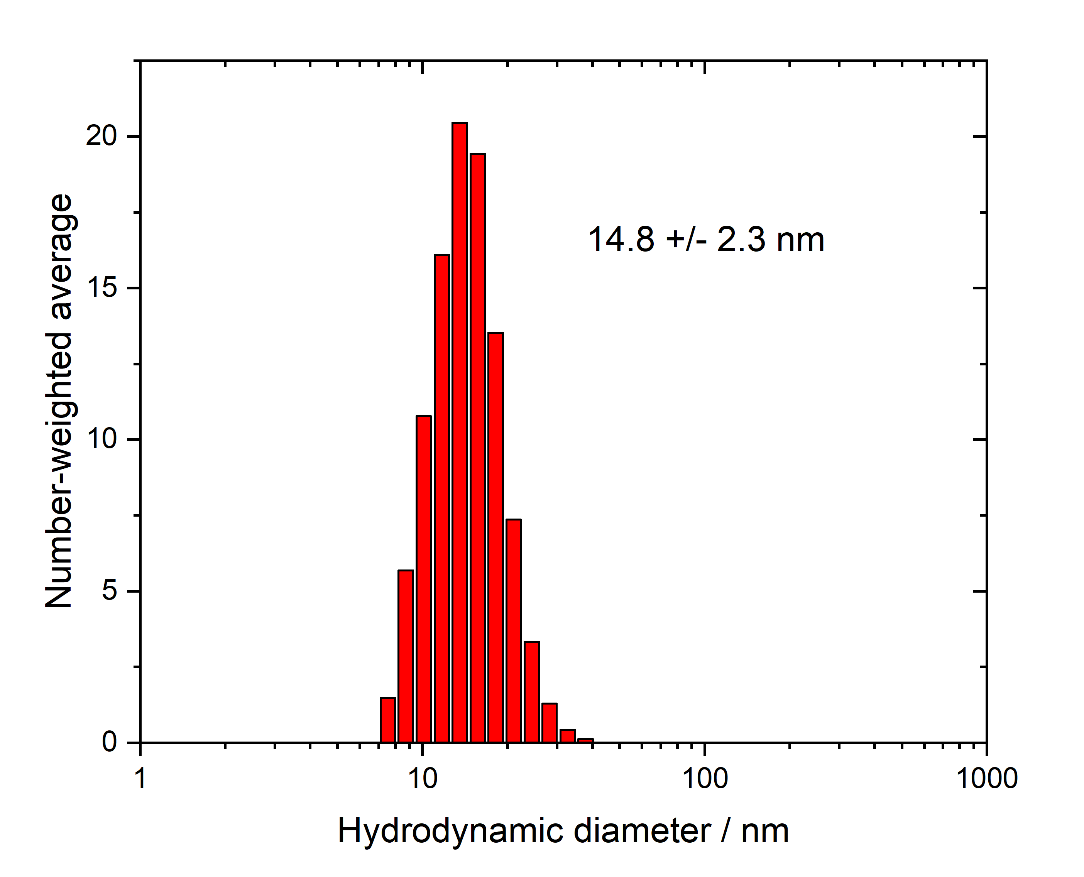


**Figure S3.** Number-weighted size distribution from DLS.

Chemical analysis of the CdSe/CdS QDs with STEM-EDX

**
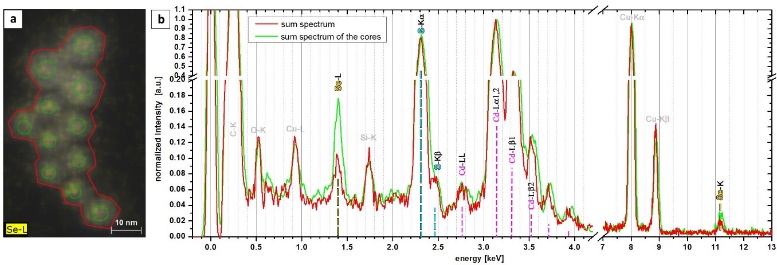
Figure S4.** EDX spectra of the CdSe/CdS nanoparticles.

a) Selenium distribution within the marked areas in which the individual point spectra were cumulated
b) EDX sum spectra within the core region (green) and the whole nanoparticles (red). Both spectra were normalized to the Cd-L edge.

Quantitative analysis of the EDX sum spectra

| Region | Cd | Se | S |
| --- | --- | --- | --- |
| core areas (green) | 55.1 +- 2.0 at% | 3.3 +- 1.8 at% | 41.6 +- 2.4 at% |
| entire nanoparticles (red) | 53.5 +- 2.0 at% | 2.1 +- 1.8 at% | 44.4 +- 2.4 at% |


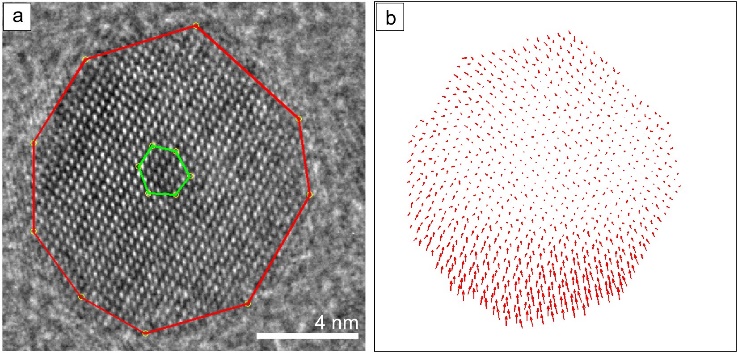


**Figure S5.** Determination of the coherence and tension of the CdSe/CdS nanoparticles using the peak finding method performed with the software ImageEval [**a**]).
a) HRTEM image of a representative CdSe/CdS nanoparticle (red: region of interest (ROI); green: reference area (core area)); b) Displacement map.

**Core/shell-structure from TEM**


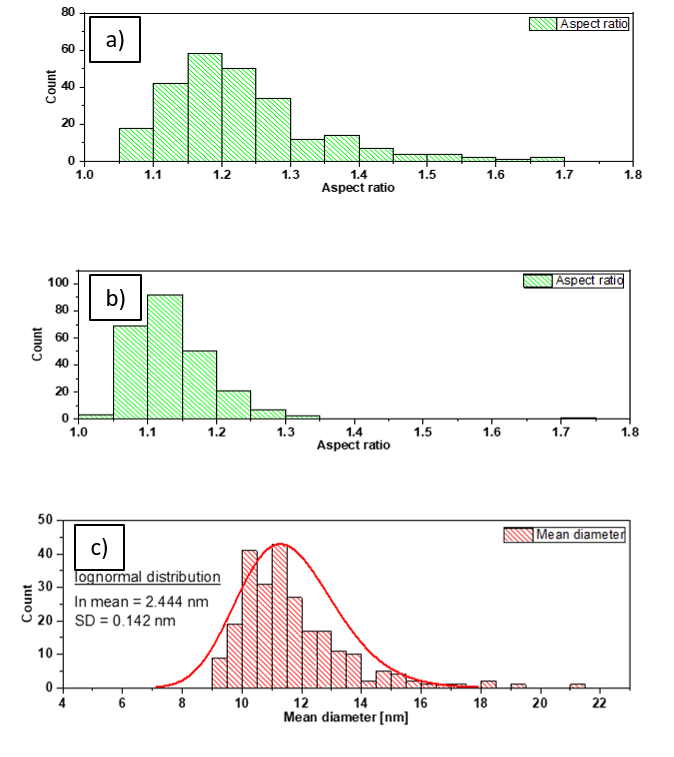


**Figure S6.**

1. Histogram of the aspect ratio of the cores
2. Histogram of the aspect ratio of the total nanoparticles
3. Histogram of the average diameter of the total nanoparticles

| 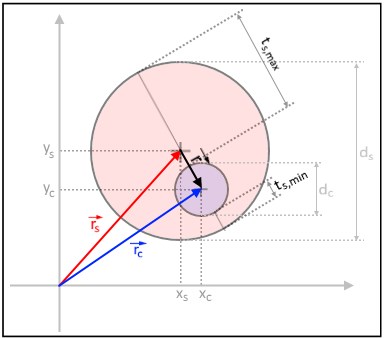 |
| --- |
| **Figure S7.** Sketch for determining the shell thickness (blue: core (c), red: shell (s)) |

Based on the parameters extracted during particle analysis and assuming that both the nanoparticles themselves and the core are spherical, the shell thickness of each individual nanoparticle was determined. Figure S8 sketches the mathematical procedure. Index c stands for core and index s for shell. First, the distance vector $\vec{r}$ between the center of gravity of the core and the center of gravity of the nanoparticle was calculated as follows:

$\vec{r}= \vec{r_{c}}$ - $\vec{r_{s}}= \left[ \begin{matrix} x_{c} \\ y_{c} \end{matrix} \right] - \left[ \begin{matrix} x_{s} \\ y_{s} \end{matrix} \right]$

Then it was checked whether the center of gravity of the core lies within shell area. If this is the case, the core and the shell were assigned to the same particle. Finally, the maximum and minimum shell thickness could be determined from simple geometric considerations as follows:

$t_{s,min}= \frac{1}{2} d_{s} -\frac{1}{2} d_{c} -\left| \vec{r} \right|$

$t_{s,max}= \frac{1}{2} d_{s} -\frac{1}{2} d_{c}+\left| \vec{r} \right|$

**Core/shell-structure from XPS**


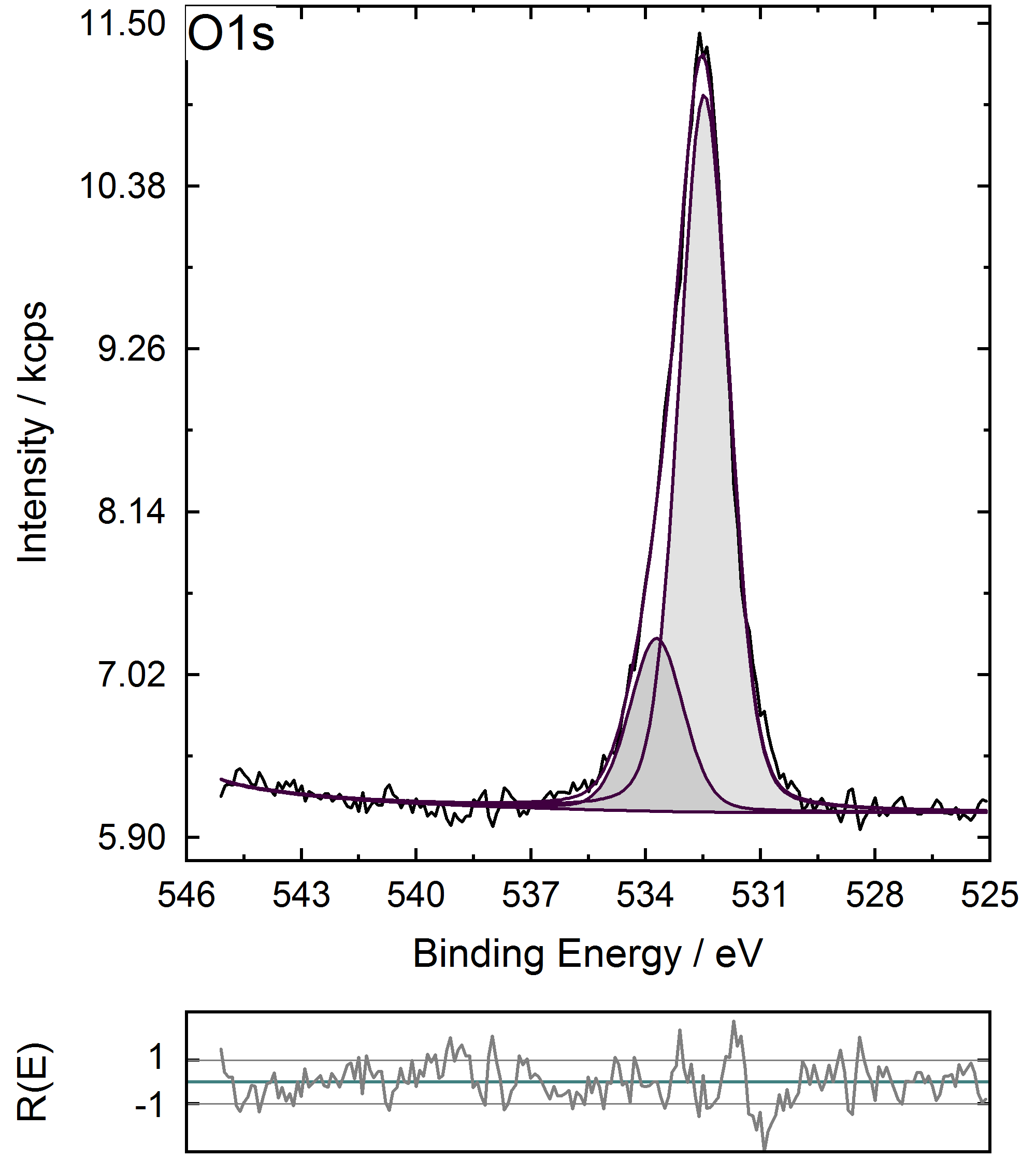

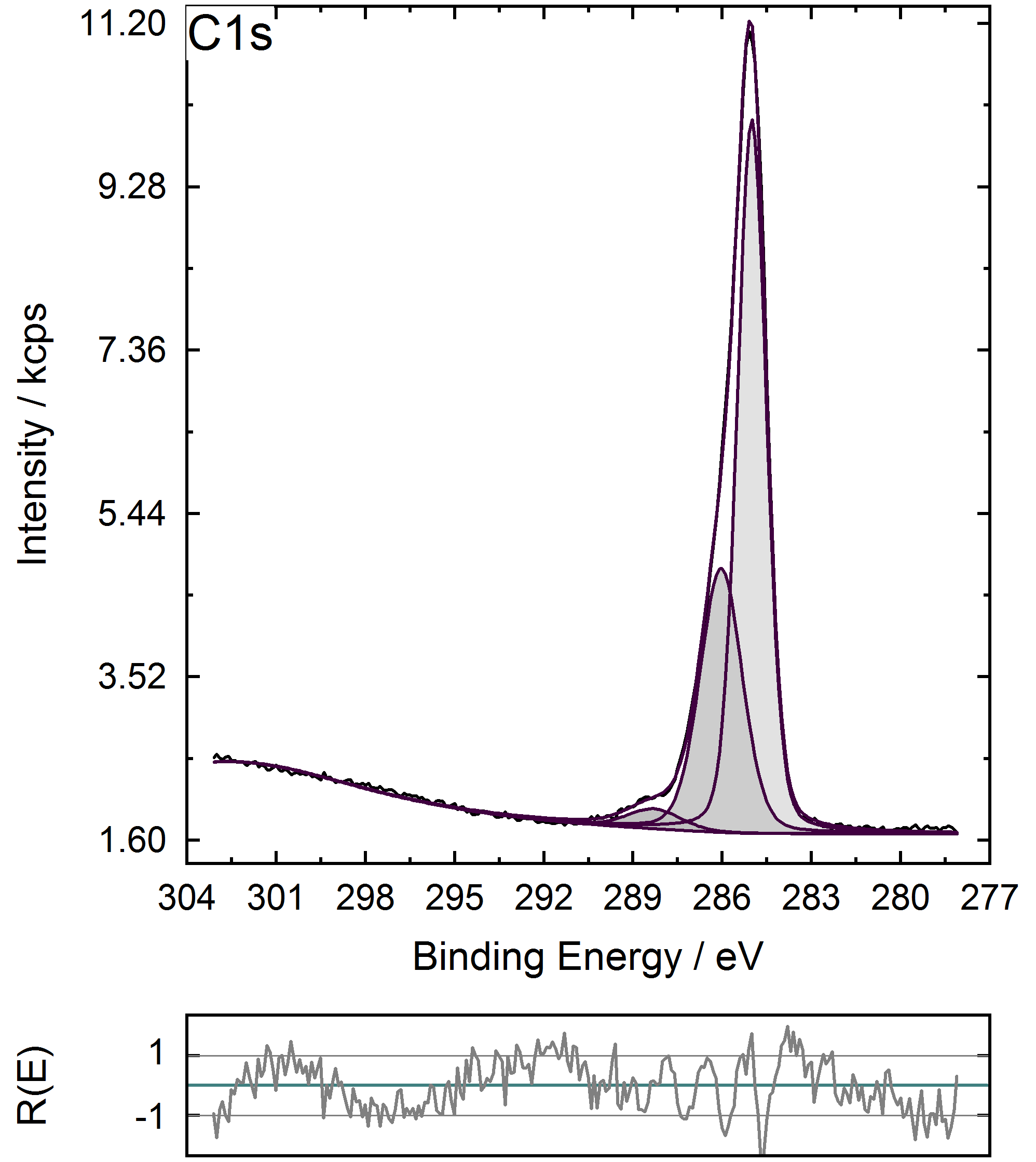


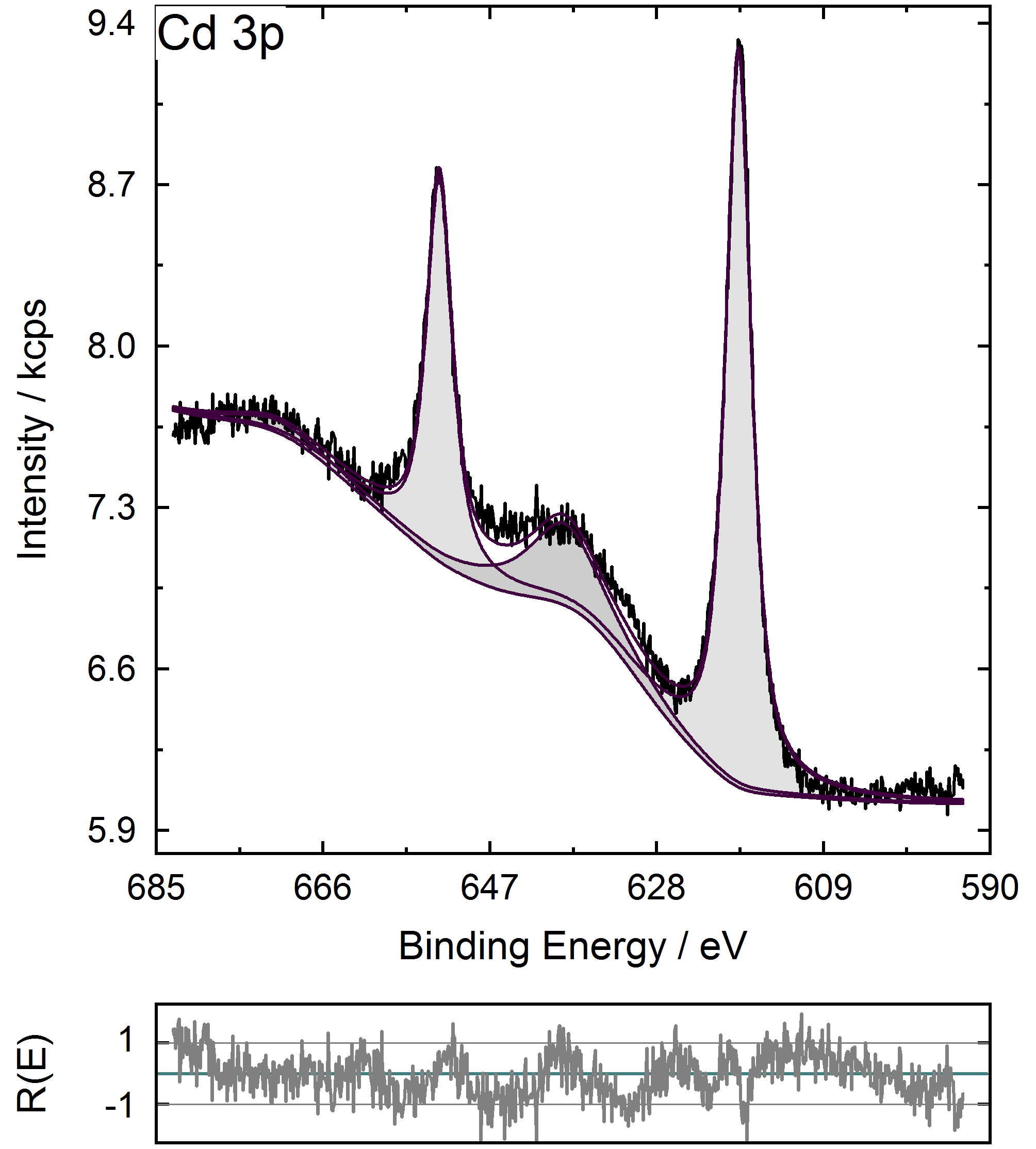

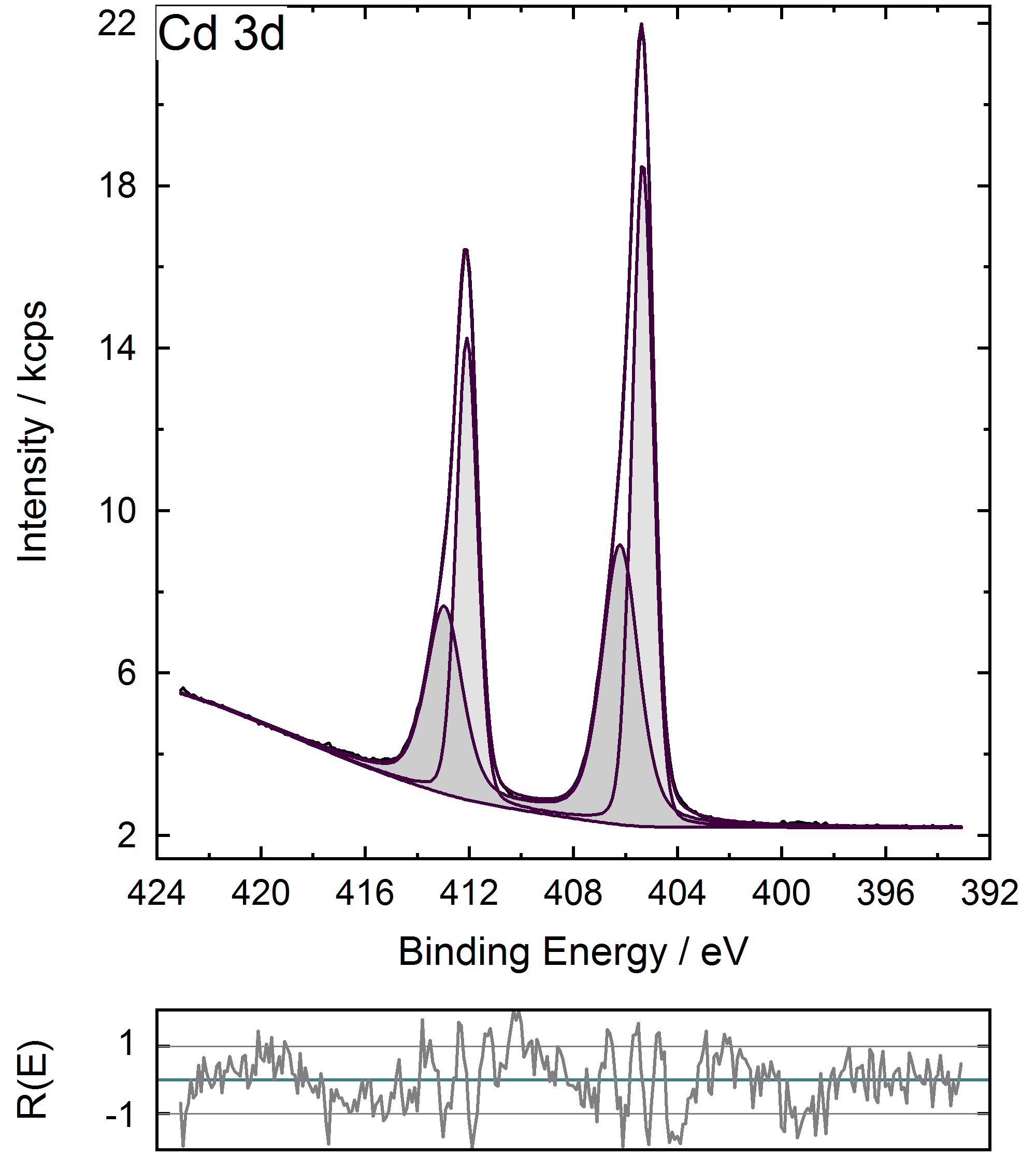


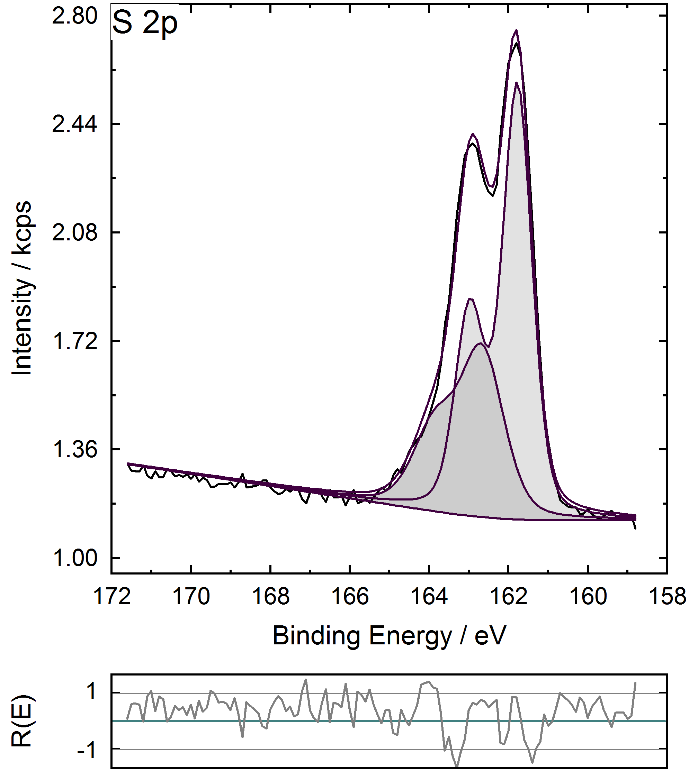

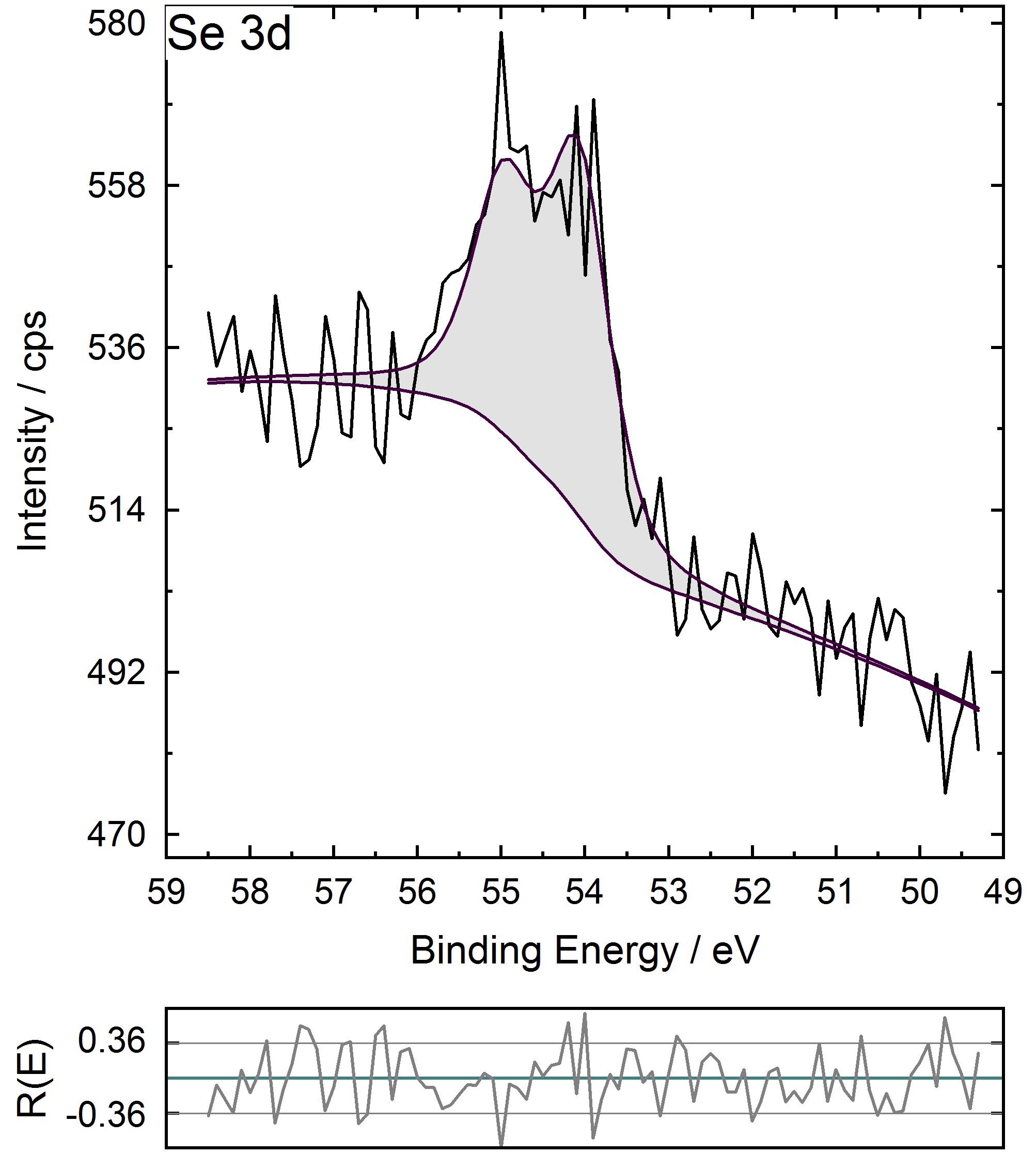


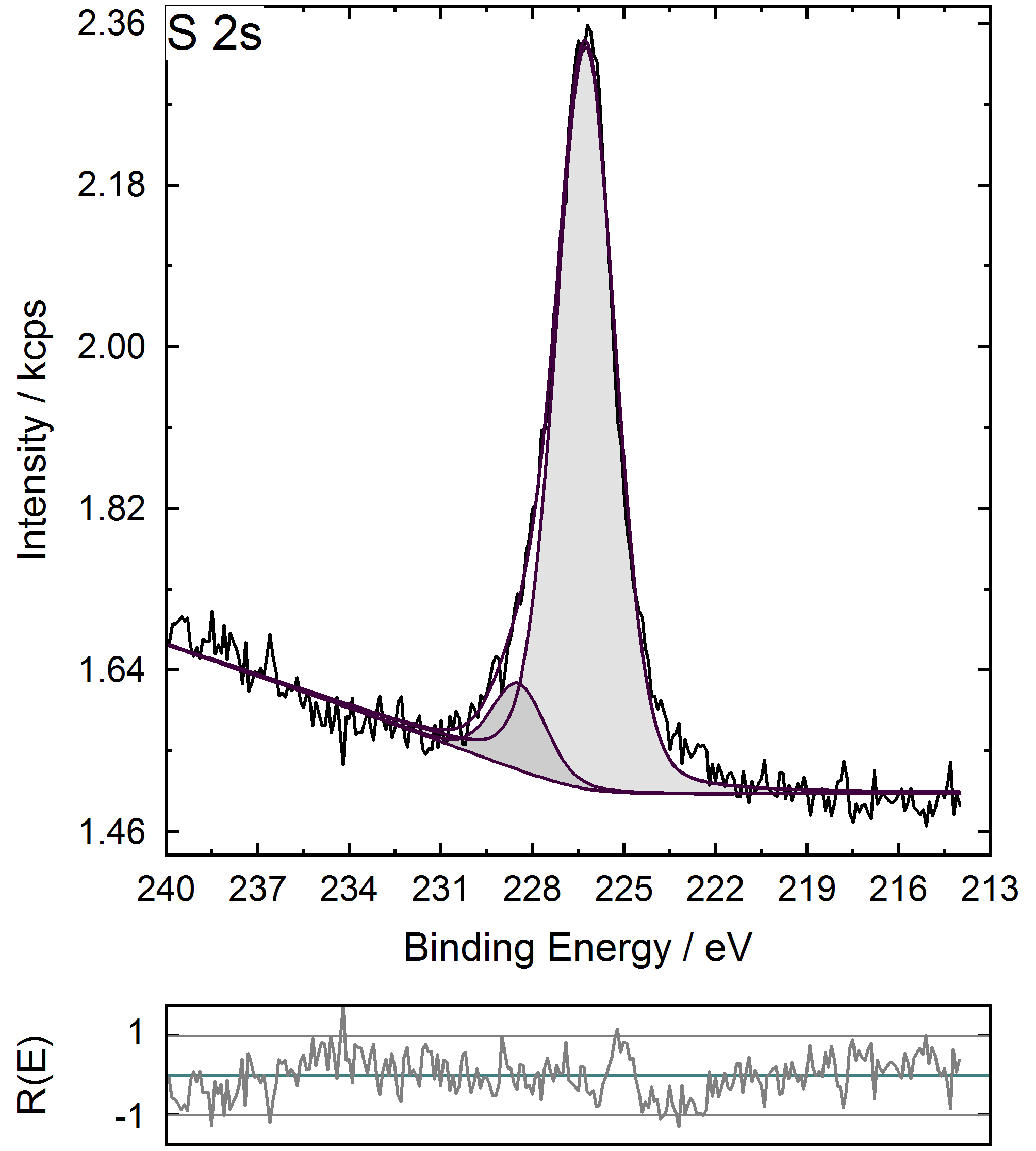


**Figure S8.** High-resolved photoelectron spectra.


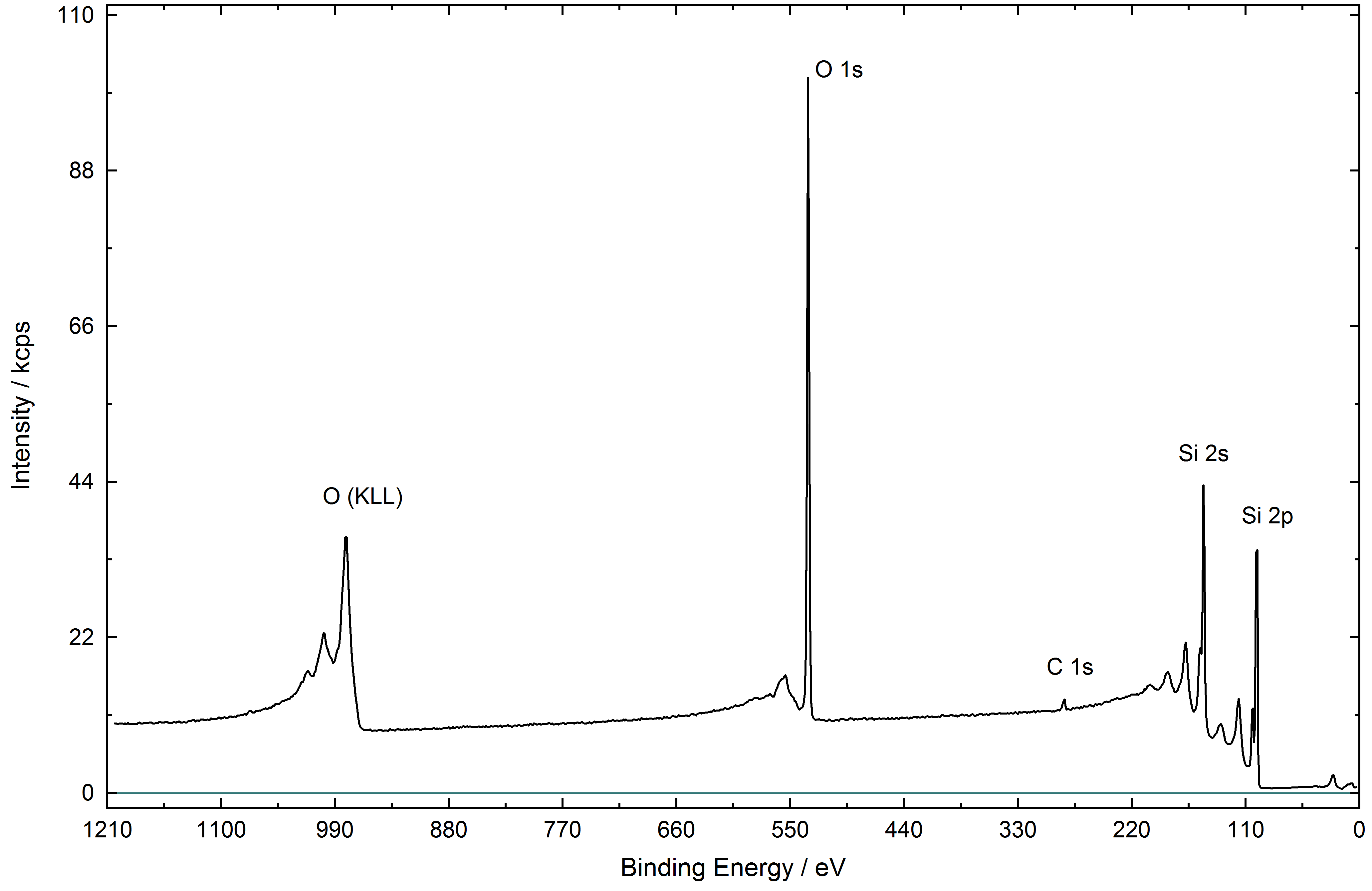


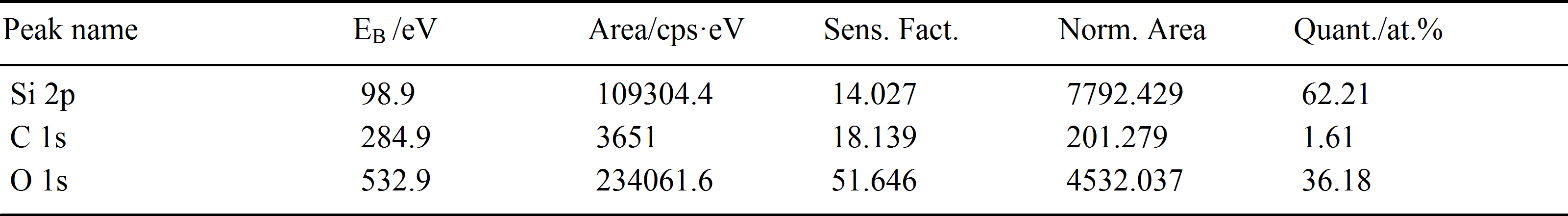


**Figure S9.** Survey spectrum of an UV/ozone cleaned pure Si wafer. The quantification was done with UNIFI 2018 using Scofield factors, inelastic mean free paths and the intensity-energy response function of the spectrometer. As model a semi-infinite sample with a homogeneous distribution was used.

**Fig. 8** Simulated intensity ratios S 2p / Se 3d for different thicknesses of an assumed intermixing zone of 0 nm and 4 nm. The chosen values of the CdSe core, the CdS shell, and the Cd2SeS intermixing zone are given for results without intermixing (top right) and with an intermixing zone of 4 nm (bottom left). The particle sizes without ligand shell are also given (top left). The experimental intensity ratio obtained from the XPS spectrum is given with lines revealing the respective uncertainty range (confidence level of 95%).


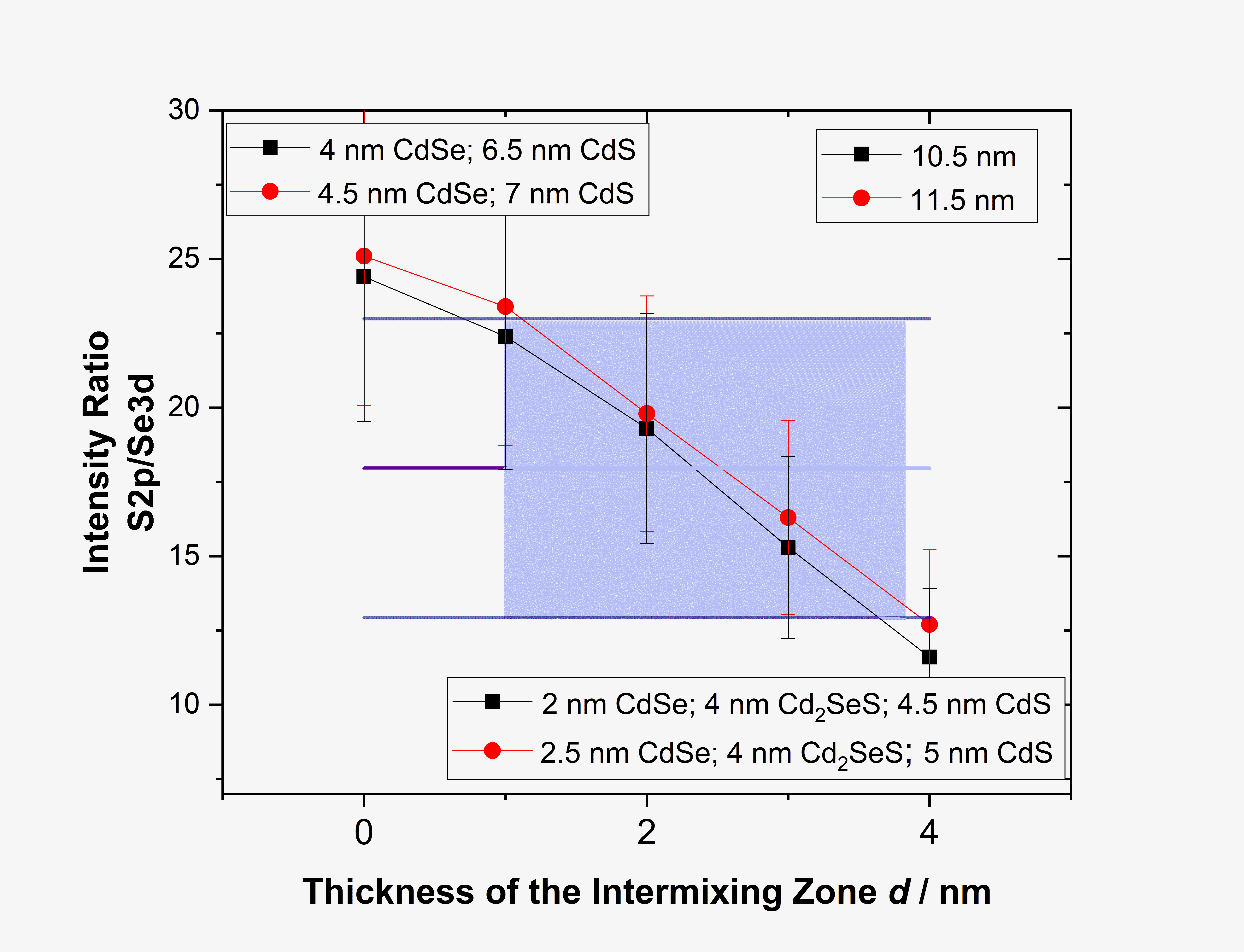


**Figure S10.** Simulated intensity ratios S 2p / Se 3d for different thicknesses of an assumed intermixing zone of 0 nm and 4 nm. The chosen values of the CdSe core, the CdS shell, and the Cd2SeS intermixing zone are given for results without intermixing (top right) and with an intermixing zone of 4 nm (bottom left). The particle sizes without ligand shell are also given (top left). The experimental intensity ratio obtained from the XPS spectrum is given with lines revealing the respective uncertainty range (confidence level of 95%).

| Peak Name | E_B_ / eV | area / cps*eV | IERF | Corr. Area / cps*eV |
| --- | --- | --- | --- | --- |
| Cd 3d | 405.1 | 290342.6 | 0.959 | 302755.0 |
| S 2s | 226.1 | 15361.5 | 0.860 | 17862.2 |
| S 2p | 162.1 | 17582.0 | 0.815 | 21572.9 |
| Se 3d | 55.1 | 857.6 | 0.714 | 1201.2 |
| O 1s | 532.1 | 70323.3 | 1.032 | 68142.7 |
| C 1s | 285.1 | 98667.6 | 0.894 | 110366.0 |
| Si 2p | 103.1 | 14036.4 | 0.765 | 21284.9 |

**Table S1.** Areas of the peaks corrected with the intensity – energy response function (IERF) of the spectrometer. These values were used for the calculation with SESSA. The estimated uncertainty is up to 10% with a confidence level of 95%.

**Table S2.** Input Parameters for SESSA and Mean Free Paths (IMFP: Inelastic Mean Free Path; EMFP: Elastic Mean Free Path)

| **Input-Parameters: single-nanoparticle model** | |  |  |
| --- | --- | --- | --- |
|  | **density / g/cm^3^** |  |  |
| **CdSe** | 5.81 |  |  |
| **CdS** | 4.82 |  |  |
| **C18H35NH2** | 0.813 |  |  |
|  |  |  |  |
|  | **Peak Position/ eV** | **Anisotropy β** |  |
| *C1s* | 1202.4 | 2.00 |  |
| *Cd 3d 3/2* | 1074.7 | 1.21 |  |
| *Cd 3d 5/2* | 1081.4 | 1.21 |  |
| *S 2s* | 1255.7 | 2.00 |  |
| *S 2p 1/2* | 1323.0 | 1.15 |  |
| *S2 p 3/2* | 1324.1 | 1.15 |  |
| *Se 3d 3/2* | 1431.1 | 1.07 |  |
| *Se 3d 5/2* | 1432.0 | 1.07 |  |
|  |  |  |  |
| **IMFP / Å** | *in C18H35NH2* | *in CdS* | *in CdSe* |
| *C1s* | 35.00 |  |  |
| *Cd 3d 3/2* | 31.98 | 21.24 | 20.27 |
| *Cd 3d 5/2* | 32.14 | 21.34 | 20.37 |
| *S 2s* | 36.25 | 23.94 |  |
| *S 2p 1/2* | 37.82 | 24.93 |  |
| *S2 p 3/2* | 37.84 | 24.95 |  |
| *Se 3d 3/2* | 40.31 | 26.50 | 25.28 |
| *Se 3d 5/2* | 40.33 | 26.52 | 25.29 |
|  |  |  |  |
| **EMFP / Å** | *in C18H35NH2* | *in CdS* | *in CdSe* |
| *C1s* | 56.44 |  |  |
| *Cd 3d 3/2* | 51.19 | 13.85 | 13.50 |
| *Cd 3d 5/2* | 51.37 | 13.89 | 13.54 |
| *S 2s* | 58.67 | 15.07 |  |
| *S 2p 1/2* | 61.48 | 15.51 |  |
| *S2 p 3/2* | 61.52 | 15.52 |  |
| *Se 3d 3/2* | 65.99 | 16.20 | 15.62 |
| *Se 3d 5/2* | 66.03 | 16.21 | 15.63 |

**Table S3.**  Influence of different ligand shell thicknesses d_OA_ on the S2p/Se 3d ratio. As an example, the results for a particle with 5 nm CdSe core and 3.25 nm CdS shell are presented (11.5 nm diameter of the CdSe-CdS particle).

| **Thickness of organic ligand shell d_OA_** | **C1s/Cd3d** | **S2p/Se3d** |
| --- | --- | --- |
| 1.5 nm | 0.26 | 18.20 |
| 1.8 nm | 0.33 | 17.58 |
| 2.0 nm | 0.41 | 17.36 |

**Table S4.** Influence of oleyl amine and oleic acid on the S2p/Se3d ratio.

As an example, the results for a particle with 5 nm CdSe core and 3.25 nm CdS shell are presented (11.5 nm diameter of the CdSe-CdS particle). 1.8 nm organic shell was assumed.

| **Kind of organic shell** | **Density / g/cm^3^** | **C1s/Cd3d** | **S2p/Se3d** |
| --- | --- | --- | --- |
| Oleyl amine | 0.813 | 0.33 | 17.58 |
| Oleic acid | 0.895 | 0.34 | 17.53 |
